# Exploring Mechanisms of Quantitative Resistance to *Leptosphaeria maculans* (Blackleg) in the Cotyledons of Canola (*Brassica napus*) Based on Transcriptomic and Microscopic Analyses

**DOI:** 10.3390/plants9070864

**Published:** 2020-07-08

**Authors:** Michelle Hubbard, Chun Zhai, Gary Peng

**Affiliations:** 1 Agriculture and Agri-Food Canada, Swift Current Research and Development Centre, Swift Current, SK S7N 0X2, Canada; michelle.hubbard@canada.ca; 2 Agriculture and Agri-Food Canada, Saskatoon Research and Development Centre, Saskatoon, SK S7N 0X2, Canada; chun.zhai@canada.ca

**Keywords:** *Leptosphaeria maculans*, canola, Phoma stem canker, genetic resistance, transcriptome analysis, RNA-seq, ROS

## Abstract

Using resistant cultivars is a common approach to managing blackleg of canola/rapeseed caused by *Leptosphaeria maculans* (*Lm*). Quantitative resistance (QR), as opposed to major-gene resistance, is of interest because it is generally more durable, due to its multi-genetic basis. However, the mechanisms and genes underlying QR are mostly unknown. In this study, potential QR modes of action in “74-44 BL” was explored. This Canadian canola cultivar showed moderate but consistent race-nonspecific resistance at the cotyledon and adult-plant stages. A susceptible cultivar, “Westar”, was used as a control. After inoculation, the lesions developed more slowly on the cotyledons of 74-44 BL than those of Westar. We used RNA sequencing (-RNA-seq) to identify genes and their functions, putatively related to this resistance, and found that genes involved in programmed cell death (PCD), reactive oxygen species (ROS), signal transduction or intracellular endomembrane transport were most differentially expressed. ROS production was assessed in relation to *Lm* hyphal growth and lesion size; it occurred beyond the tissue colonized by *Lm* in 74-44 BL and appeared to trigger rapid cell death, limiting cotyledon colonization by *Lm*. In contrast, *Lm* grew more rapidly in Westar, often catching up with the ring of ROS and surpassing lesion boundaries. It appears that QR in 74-44 BL cotyledons is associated with limited colonization by *Lm* possibly mediated via ROS. The RNA-seq data also showed a link between ROS, signal transduction, and endomembrane vesicle trafficking, as well as PCD in the resistance. These results provide a starting point for a better understanding of the mechanisms behind QR against *Lm* in canola.

## 1. Introduction

Canola or rapeseed (*Brassica napus* L.) is an economically important oilseed crop cultivated worldwide. Blackleg, caused by *Leptosphaeria maculans* (*Lm*) Ces. and de Not., is a serious disease of canola, especially in Australia, Europe, and Canada [1]. Genetic resistance is a cornerstone of blackleg management and is usually classified as either qualitative or quantitative. The former is controlled by single resistance (R) genes, while the latter is often, though not always, polygenic [2,3]. While many of the R genes have been identified [4,5,6,7,8,9], quantitative resistance (QR) is not well understood. QR can be attributed to multiple genomic regions in *B. napus* [10], with many of the same loci found in multiple canola cultivars [11]. QR to blackleg in canola is believed to be expressed primarily in adult plants. However, “74-44 BL”, a Canadian canola cultivar used in this study, has consistently shown QR to stem canker in adult canola, as well as to infection in cotyledons by *Lm* [12]. Poland et al. [13] postulated that plant QR might be due to weaker versions of R genes, alterations in plant morphology or development, phytoalexin production, variants of innate immunity, or signal transduction associated genes. QR in canola to *Lm* might also be attributed to uncharacterized R genes [11,14,15,16,17].

RNA sequencing (RNA-seq) has provided valuable insights into the interactions between canola and blackleg in the initial stages of cotyledon infection in the absence of genetic resistance [18], in canola with and without major resistance genes [19,20,21], as well as the genes that are potentially involved in other plant–pathogen interactions. For example, Hao et al. [22] used RNA-seq to explore QR to rust in wheat. In addition, Joshi et al. [23] used RNA-seq to identify genes involved in resistance to *Sclerotinia* in *B. napus*. Haddadi et al. [18] found that, in the absence of any known resistance, genes related to initial lignin biosynthesis, as well as those involved in biosynthesis, breakdown of glucosinolates and cell surface receptors (PAMP and effector recognition) were upregulated. In contrast, transcription factors, proteases, and protease inhibitors, peroxidases, and chitinases were expressed to a lesser extent within blackleg lesions. However, it is not known if any of these host responses can be induced in seedlings. Larkan et al. [14] found evidence that a cluster of receptor-like kinases could be involved in the QR of adult canola plants against blackleg. Consistent with this finding, Haddadi et al. [18] reported that one of the cell surface receptors expressed differentially in blackleg-infected seedlings was also a receptor-like kinase. Additional studies have identified regions in the *B. napus* genome that are potentially involved in QR to *Lm* [10,11,15,16,17,24]. It is therefore useful to explore the modes of action for QR against *Lm*.

Next generation sequencing approaches might help relate phenotypic observations, such as those obtained from microscopy, to molecular mechanisms. Fluorescent microscopy of proteins tagged with fluorophores, such as green fluorescent protein (GFP), provides valuable information about plant colonization by microbes, including the canola-blackleg pathosystem [12,25]. Similarly, imaging of the ROS in canola cotyledons can complement RNA-seq data. This study presents data on *Lm* colonization in cotyledons of Westar (susceptible) and 74-44 BL (expressing QR and carrying two specific *R* genes in a hybrid background) inoculated with a GFP-expressing *Lm* isolate. This work also aimed to explore the genes that are differentially expressed between canola cultivars in seedlings, in an attempt to gain insights into the potential mechanisms of QR in the resistant cultivar 74-44 BL.

## 2. Results

### 2.1. Infection Symptoms and Lm Hyphal Growth in the Cotyledons of Susceptible and QR Canola Cultivars

**Figure 1:**
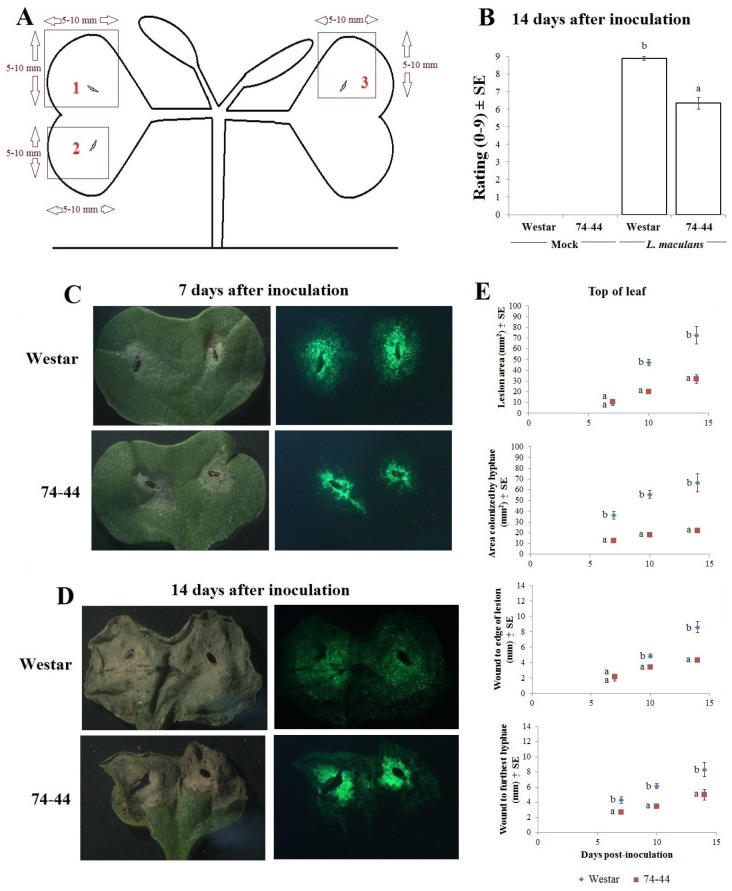
Approximate size and location of the cotyledon samples taken for RNA-seq analysis (**A**); subsamples (labeled 1, 2 and 3 in red) were taken from three individually-inoculated cotyledon lobes of each plant. Infection severity (0–9 scale) in cotyledons of Westar and 74-44 BL at 14 dpi (**B**), grown with the plants used for RNA-seq analysis. Lesions and GFP-expressing *Lm* hyphal growth in Westar and 74-44 BL cotyledons at 7 (**C**) and 14 (**D**) dpi. The lesion size, area colonized by *Lm* hyphae, distance from the center of pricking inoculation to the furthest edge of the lesion, or to the furthest hyphal tips (**E**). Bars or data points with the same letter at a given time point in the same panel are not different (*t*-test, *p* > 0.05).

Among the seedlings inoculated and grown in parallel with those used for RNA-seq, Westar (susceptible) showed higher infection ratings than 74-44 BL, at 14 days post inoculation (dpi) (Figure 1B). However, in separate experiments, the appearance and size of lesions, as well as the distance from the inoculation wound (pricking) to lesion edge, were similar between the two cultivars at 7 dpi. The area colonized by the *Lm* hyphae and the distance from the inoculation center to the most distal hyphal tips were greater in Westar (Figure 1C,E). By 10 and 14 dpi, all measurements had become greater in Westar than 74-44 BL (Figure 1D,E).

### 2.2. RNA-Seq Analyses

#### 2.2.1. Expression of *L. maculans* Genes in Inoculated *B. napus*

**Figure 2:**
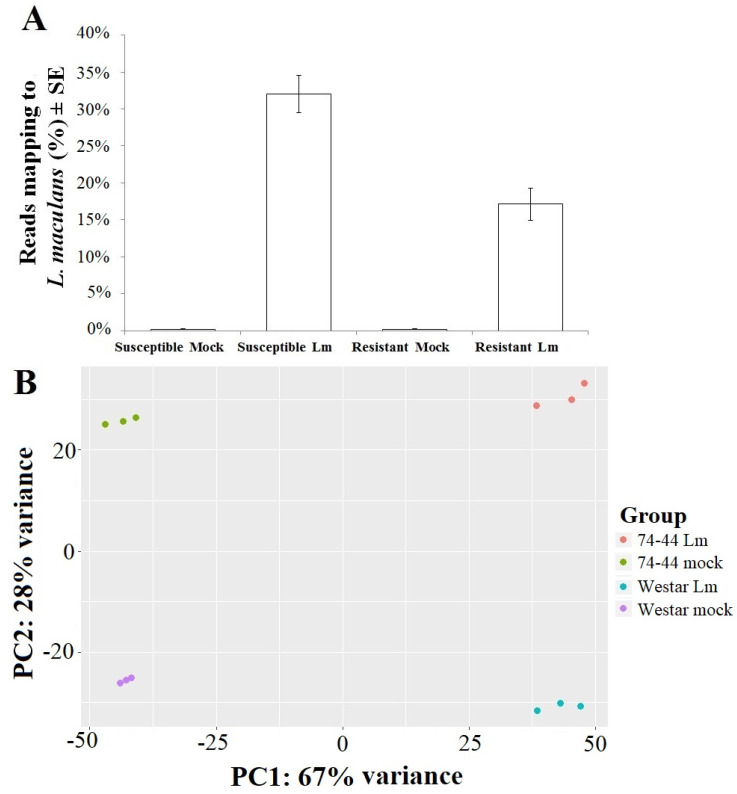
Percent of reads mapped to the *Lm* genome (**A**) and principle-component-analysis (PCA) plot, produced by DESeq2 (**B**).

Three libraries (replicates) were produced for each of the four treatments, i.e., Westar and 74-44 BL with mock and *Lm* inoculation, respectively. A total of twelve libraries were used for RNA-seq. Approximately 14.1–17.8 million paired-end reads were obtained from each library (Supplementary Table S1). When annotated against *Lm* genome, a higher percentage of reads was mapped for the inoculated Westar, as compared to the inoculated 74-44 BL (Figure 2A). Principle-component analysis (PCA) indicated that the treatments grouped tightly together in terms of their alignment to the *Lm* genome (Figure 2B). Using the criteria of adjusted *p* value ≤ 0.05 and log_2_ fold change ≥ 2, only 16 differentially expressed genes (DEG) of *Lm* were identified between inoculated Westar and 74-44 BL, with three DEGs up-regulated and thirteen down-regulated in the 74-44 BL, relative to those in Westar (Supplementary Tables S2 and S3).

The *Lm* genes generally were expressed at low levels; only eight of them showed a base-mean expression value over 10,000 (14,346 to 40,534), three between 1000 and 9999, 85 between 100 and 999, and 152 between 50 and 99. There were a total of 12,119 *Lm* genes with non-zero expression values, the vast majority of which had base-means under 50.

The three upregulated *Lm* DEGs in inoculated 74-44 BL, had sequence similarities to genes encoding a short-chain dehydrogenase/reductase, a pyoverdine biosynthesis, and a hypothetical protein, respectively. Pyoverdine is a siderophore biosynthesized by *Pseudomonads* [26]. Zwiers et al. [27] found a gene encoding an ABC-transporter with a pyoverdine biosynthesis motif in the fungus *Mycosphaerella graminicola*. ABC-transporters can play a role in the virulence of fungal pathogens [28,29]. It is unclear what role, if any, these *Lm* DEGs played during the infection of 74-44BL.

#### 2.2.2. Expression of the *B. napus* Genes

**Figure 3:**
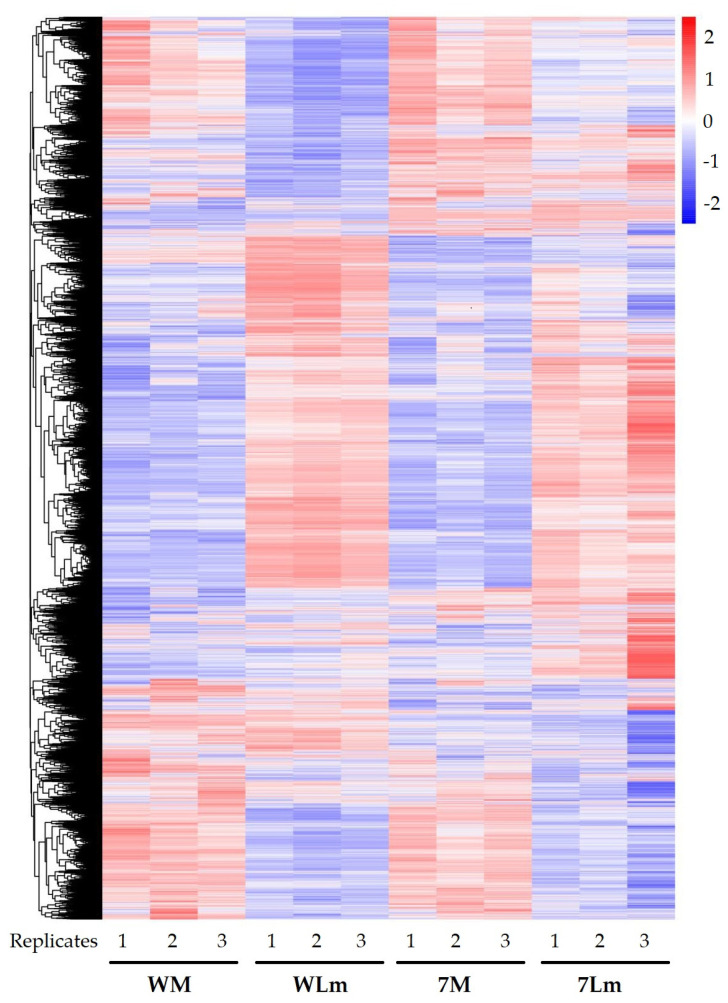
Cluster heatmap of the expressed genes across all samples. Z-score transformation was performed for each gene. Genes with similar expression pattern were clustered together. Relative expressions were scaled from red (high expression) to blue (low expression). Each column represents a sample (replicate), and each row represents a gene. WM: Westar mock, WLm: Westar *Lm*, 7M: 74-44 BL mock and 7Lm: 74-44 BL *Lm*.

A heatmap based on the level of relative gene expression showed similar patterns among three replicates within a treatment (Figure 3). Similarities, as well as differences, were observed between inoculated Westar and 74-44 BL, and also between two mock inoculations.

##### Upregulated *B. napus* Genes in Inoculated 74-44BL

**Figure 4:**
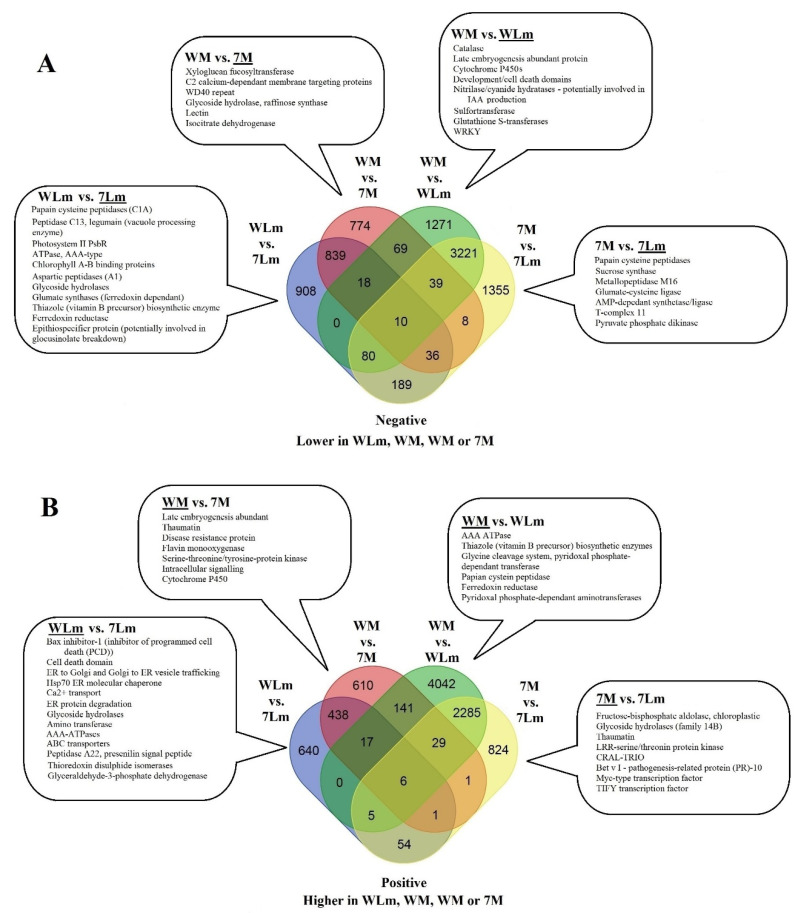
Venn diagrams [31] (of upregulated (**A**) and downregulated (**B**) differentially expressed genes (DEGs) between *L. maculans* inoculated Westar (WLm) and 74-44 BL (7Lm), mock-inoculated Westar (WM) and 74-44 BL (7M), and between mock and inoculated Westar or mock and inoculated 74-44 BL. Genes associated with the underlined treatment are upregulated significantly in the paired comparisons.

**Figure 5:**
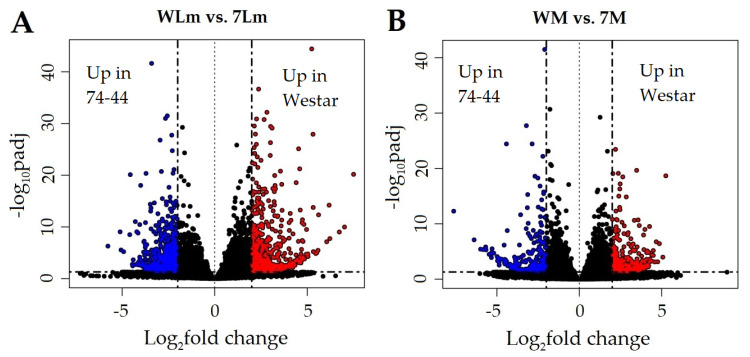
Volcano plots of DEGs between *L. maculans* inoculated Westar (WLm) and 74-44 BL (7Lm) (**A**), or between mock-inoculated Westar (WM) and 74-44 BL (7M) (**B**). Red and blue dots represent up- and downregulated DEGs, respectively, while the black dots indicate non-DEGs. X-axis: Log_2_-fold change; and Y-axis: Log_10_ adjusted *p* values.

A total of 908 genes in the *B. napus* genome were differentially upregulated in inoculated 74-44 BL, relative to those in inoculated Westar (Figure 4A). The majority of these DEGs had adjusted *p* values and log_2_-fold changes in expression relatively close to the cut-off levels (Figure 5A). Two of the DEGs showed base-mean expression levels over 10,000, six between 5000 and 9999, and 65 between 4999 and 1000. Five DEGs putatively encoding peptidases were among those with the highest scores (Supplementary Table S4). The three highest-scored DEGs, BnaA01g17570D, BnaA01g04000D and BnaA09g52180D, all encode peptidases. BnaA01g17570D, with InterPro domains, points to a cysteine peptidase belonging to family C1 (papain-like). BnaA01g04000D is a putative legumain peptidase C13, also known as a vacuole processing enzyme (VPE). BnaA09g52180D is also related to a cysteine peptidase. The DEG BnaC02g00130D potentially encodes a protease involved in RuBisCO degradation. Additionally, many chlorophyll A-B binding proteins showed high basemeans and were more highly expressed in inoculated 74-44 BL (Supplementary Table S4), such as an ATPase of AAA-type with a protein BLAST similarity to RuBisCO activase. This protein is potentially involved in endoplasmic reticulum (ER) to Golgi membrane budding.

Genes that potentially encode glycoside hydrolases, including a beta-galactosidase (BnaA04g04110D) and a pullulanase-type alpha-1,6-glucosidase (BnaA10g25820D), were upregulated in inoculated 74-44 BL, with small *p* values (Supplementary Table S4). A gene putatively encoding lactate/malate dehydrogenase (BnaC02g00740D) was also upregulated, albeit with a less significant *p* value but a higher base-mean expression than BnaA04g04110D or BnaA10g25820D (Supplementary Table S4, Figure 4). BnaA03g11710D, with a thiazole biosynthetic enzyme InterPro domain, is a DEG with a protein sequence similar to a ribulose-1,5-biphoshate synthetase.

Gene ontology (GO) term enrichment analysis of these 908 DEGs was consistent with the results described above (Supplementary Table S13, Figure 4); many of the GO terms with the lowest FDR were related to photosynthesis and light responses. Similarly, the KEGG metabolic-pathway annotation also included highly expressed DEGs linked to photosynthesis with low adjusted *p* values and extremely high log_2_-fold changes in expression (Supplementary Table S14). Furthermore, three GO terms were linked to hydrogen peroxide (HP). While none of the enriched GO terms suggested peptidase activities, the GO term with the second lowest FDR was associated with cysteine biosynthesis (Supplementary Table S13). This was consistent with the putative cysteine peptidase activity found with the DEG BnaA01g17570D (Supplementary Table S4).

#### 2.2.3. Upregulated *B. napus* Genes in Inoculated Westar

A total of 640 genes were more highly expressed in inoculated Westar, as compared to inoculated 74-44 BL (Figure 4B). These DEGs generally displayed lower expression values relative to the 908 DEGs upregulated in inoculated 74-44BL, ranging from a base-mean of 3,410 to 1.25. Only 11 DEGs showed base-means over 1000, 28 between 500 and 999, 73 between 100 and 499, and the remaining 527 were under 100. However, these upregulated DEG clusters were noticeably more above the cut-off levels (log_2_-fold change, adjusted *p* value) than those in inoculated 74-44 BL (Figure 5A).

The DEG with the highest basemean, BnaC09g20030D, showed similarity to the genes related to a Bax inhibitor-1 (Supplementary Table S13), while the DEG BnaCnng58090D, with a basemean of 2354, was linked to a development/cell death domain (DCD). Other significant DEGs included BnaC08g42820D, which was associated with a heat shock protein 70, as well as BnaA04g06220D and BnaA09g26960D, which were linked to Sec23/Sec24 and Sec61/SecY, respectively. Sec23 and sec24 are part of the coat protein II (COPII) complex that is involved in endoplasmic reticulum (ER) to Golgi vesicle transport [30]. The DEGs BnaA08g26550D, BnaA06g05280D, BnaC06g24690D, BnaA07g09950D, and BnaCnng06680D, with basemeans ranging from 972 to 3100, appeared to be related to small GTPases (Supplementary Table S13, Figure 4A).

GO terms related to ER, ER stress, vesicle transport, and the cellular endomembrane system were enriched in *Lm*-inoculated Westar. However, none of the enriched GO terms were associated with PCD. One enriched GO term was related to the HP response (Supplementary Table S8). BnaCnng58090D did not appear to be associated with any GO terms. KEGG annotation revealed less information than the DEGs upregulated in 74-44 BL. The KEGG data did not identify a connection between the resistance and the endomembrane system or PCD (Supplementary Table S9). However, this was unsurprising since KEGG focuses on metabolic pathways. Multiple DEGs were annotated by KEGG in relation to purine metabolism.

#### 2.2.4. Upregulated *B. napus* Genes in Mock-Inoculated 74-44 BL

There were 774 genes upregulated in mock-inoculated 74-44 BL, relative to mock-inoculated Westar, including InterPro domains (BnaC08g39910D), which suggest xylogucan fucosyl-transferase activity, putative C2 calcium-dependent membrane targeting genes (BnaA09g48570D, BnaA09g05080D), and those with WD40 repeats (BnaA05g22060D, BnaC03g03670D). In addition, BnaC09g37530D showed putative glycoside hydrolase and raffinose synthase activity. A potential lectin (BnaA01g28810D) and isocitrate dehydrogenase (BnaC03g01130D) were also upregulated (Supplementary Table S14). Only one GO term, with the molecular function of histone acetyltransferase activity, was enriched among these DEGs (Supplementary Table S14), with two and four DEGs showing log_2_-fold change values < −5 or log_10_ adjusted *p* values < −20 (Figure 5B). There were only three DEGs with scores (based on expression, log_2_-fold change in expression, and adjusted *p* value) < 1 × 10^3^, with the KEGG annotation relating to fructose and mannose or starch and sucrose metabolism, or to fatty acid biosynthesis and elongations (Supplementary Table S12).

#### 2.2.5. Upregulated *B. napus* Genes in Mock-Inoculated Westar

A total of 610 genes were more highly expressed in mock-inoculated Westar, relative to the mock-inoculated 74-44 BL. Some of the highest scoring DEGs were related to late embryogenesis abundant protein (BnaA03g55320D), thaumatin (BnaC05g14950D), disease-resistance protein (BnaA09g14680D), putative flavin monoxygenase (BnaC08g41540D), serine/threonine-protein kinase (BnaC03g59870D), a phox-associated InterPro domain (BnaA03g32960D) involved in intracellular signaling pathways, and a cytochrome P450 domain (BnaCnng49630D) (Supplementary Table S13). There were only two enriched GO terms, with the molecular functions associated with ADP binding and monooxygenase activity (Supplementary Table S14). Only four and 11 of these DEGs had log_10_-adjusted *p* values < −20 and log_2_-fold changes > 5, respectively (Figure 5B). Only three DEGs involved in nitrogen metabolism, phenylpropanoid biosynthesis/glycerphospholipid, and ether lipid metabolism had scores > 1 × 10^3^ in the KEGG analysis (Supplementary Table S15).

### 2.3. HP Production in Cotyledons

RNA-seq results suggested that ROS, such as HP, might play a role in QR of 74-44 BL against *Lm*. To validate this finding, cotyledon tissues were stained with 3,3-diaminobenzidine (DAB), to quantify the area of ROS production surrounding the inoculation site.

**Figure 6:**
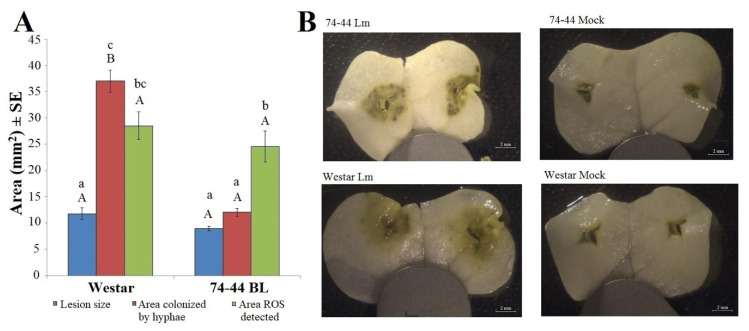
The area of visible lesions, *Lm* hyphal colonization and positive staining for HP(a ROS) in the cotyledons of Westar and 74-44 BL at 7 dpi with the *L. maculans* isolate 12CC09-GFP. Bars with the same letter of the same case are not significantly different (**A**). Capital letters indicate comparisons between the cultivars for a given parameter (Wilcoxon two-sample test, *p* ≤ 0.05). Lower case letters denote comparisons between parameters, within a cultivar (Tukey adjusted, *p* ≤ 0.05). Panel (**B**) shows the appearance of representative HP staining reactions on cotyledons.

At 7 dpi, the lesion size, extent of *Lm* hyphal colonization, and area with ROS reaction varied on inoculated cotyledons, depending on the cultivar and parameter measured. In Westar, the area colonized by hyphae (as visualized by GFP fluorescence) and the area stained positive for HP were both larger than the necrotic lesions (Figure 6A). In contrast, the lesion size and area colonized by the *Lm* hyphae were significantly smaller than the area stained for ROS in 74-44 BL. As with the results in Figure 1, the lesion size did not differ between the two cultivars at 7 dpi, while the area colonized by the *Lm* hyphae was substantially greater in Westar. The area with ROS staining did not differ between the cultivars (Figure 6B).

**Figure 7:**
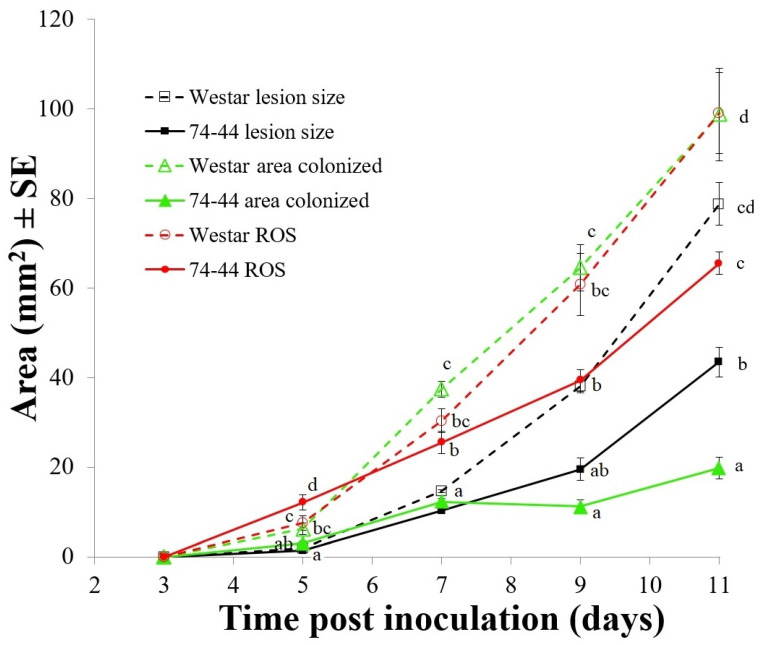
Time-series of the area of visible lesions, *Lm* hyphal colonization and positive DAB staining for HP, a ROS, in the cotyledons of Westar and 74-44 BL. Values with the same letter(s), at the same time point, did not differ (Tukey adjusted, *p* ≤ 0.05).

The HP reaction was also examined in a time-course post-inoculation, in order to obtain more detailed data than that presented in Figure 6. Most parameters measured tended to increase with time, but the responses varied between cultivars. In Westar, *Lm* colonization developed more rapidly than it did in 74-44 BL, with significant development beyond the lesions (*p* ≤ 0.05, Tukey adjusted) but generally within the boundary of DAB staining (Figure 7). In 74-44 BL, *Lm* hyphal growth was more restricted, relative to the DAB-stained areas at 5–11 dpi, and to both DAB staining and the lesion at 11 dpi (Tukey adjusted, *p* ≤ 0.05).

### 2.4. Genomic DNA Degradation as an Indicator of PCD

**Figure 8:**
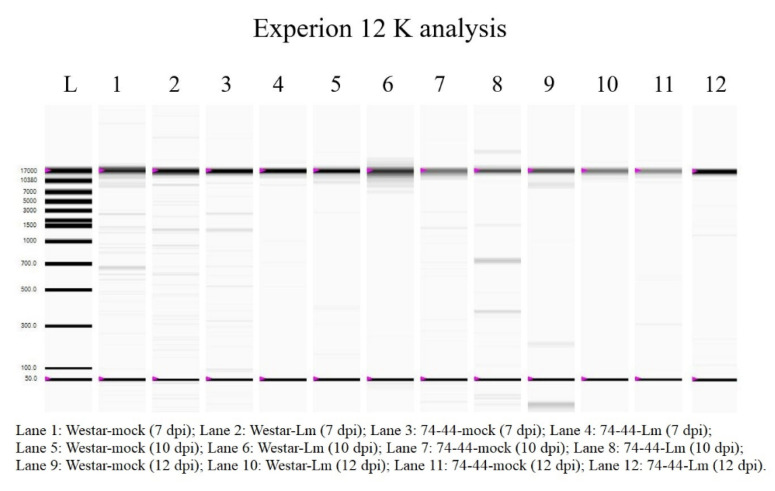
Genomic DNA from mock-inoculated Westar (Westar-mock), Westar inoculated with *L. maculans* (Westar-Lm), mock-inoculated 74-44 BL (74-44-mock), and 74-44 BL inoculated with *L. maculans* (74-44-Lm), separated on an Experion 12K chip in order to assay genomic DNA degradation as a marker of programmed cell death.

As RNA-seq showed that PCD could also play a role in QR in 74-44 BL, degradation of genomic DNA was examined as a proxy for PCD. However, no apparent difference in genomic DNA degradation was observed between the inoculated Westar and 74-44 BL, based on either agarose gel electrophoresis or Experion 12K (Figure 8).

### 2.5. Impact of Protease Inhibitors on Lm Infection of Cotyledons

**Figure 9:**
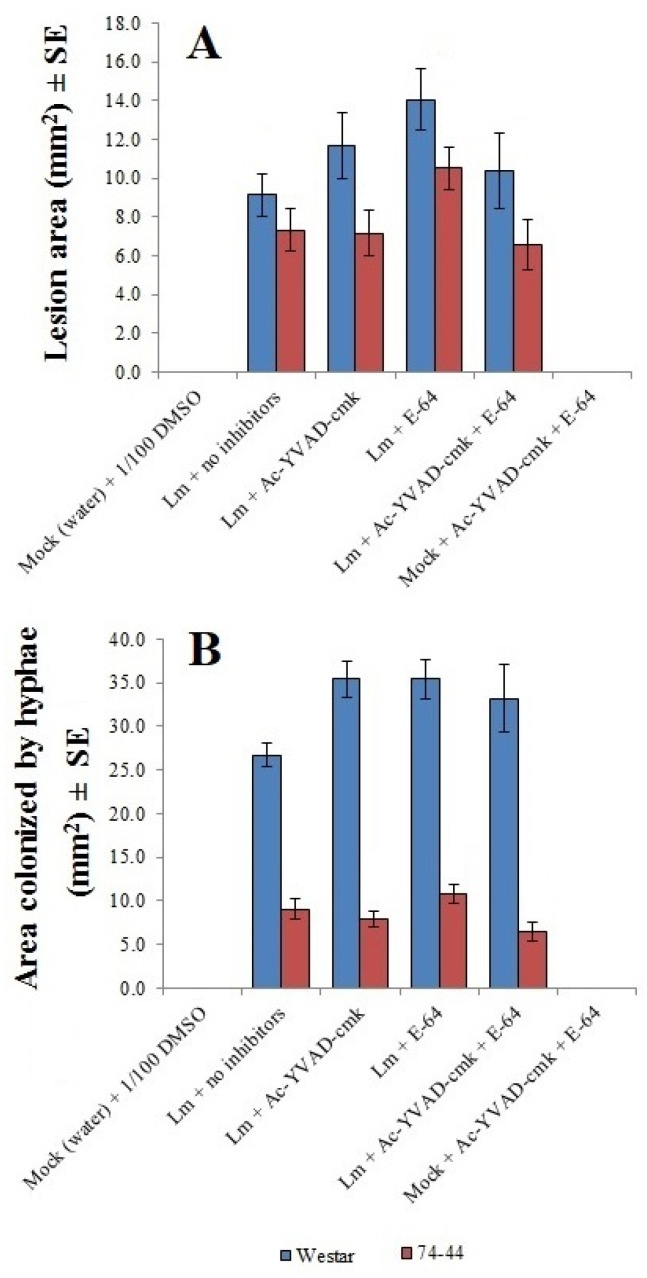
Impact of protease inhibitors on lesion size (**A**) and area colonized by *Lm* hyphae (**B**) in the cotyledons of Westar and 74-44 BL at 7 dpi.

Results from RNA-seq also led to the hypothesis that proteases could contribute to QR in 74-44 BL. Several protease inhibitors, when applied in liquid to the surface of cotyledons of Westar or 74-44 BL, failed to show any significant impact on either the lesion size or the extent of *Lm* hyphal colonization, although the latter was consistently greater in Westar (Figure 9).

## 3. Discussion

QR to *Lm* was expected to be more prominent in adult canola/rapeseed plants than in seedlings [2]. However, this study demonstrated a quantitative reduction of *Lm* infection in cotyledons of 74-44 BL, in the absence of major-gene involvement, by using an isolate without an *Avr* gene corresponding to any of the *R* genes in this resistant cultivar. Specifically, more restricted host tissue colonization and lesion development were observed on 74-44 BL, relative to Westar. Earlier studies [12,32] also found consistent QR with 74-44 BL in stems, as well as in cotyledons, against multiple *Lm* races, confirming the race-nonspecific nature of resistance. This study also found that the *Lm* hyphal growth often extended beyond the borders of visible lesions in Westar, while the growth was consistently restricted within the lesion in 74-44 BL. Huang et al. [25,33] also measured QR to *Lm* growth in young *B. napus* plants and in some cases, found that this restricted early growth correlated with reduced blackleg in more mature plants [33]. They also found partial overlap in quantitative trait loci (QTL) contributing to *Lm* resistance at both young and adult plant stages. RNA-seq was used to explore genes potentially involved in the seedling–stage QR in 74-44 BL as a first step to understanding the molecular mechanisms.

Many of the highly upregulated genes in Westar relate to the control of PCD, endomembrane vesicle trafficking between the ER and Golgi, as well as molecular chaperones, cation transporters, protein glycosylases, and degradation enzymes (Supplementary Table S13). The GO terms enriched in these DEGs also support a role with endomembrane vesicle transport (Supplementary Table S13). The upregulation of BnaCnng58090D (Supplementary Table S13), a gene with sequence similarity to a development/cell death (DCD) domain, could potentially stimulate a hypersensitive reaction, a form of PCD in plants [34], in response to infection. Other upregulated genes with putative roles in endomembrane transport to/from the ER are potentially related to ER stress, which can also trigger DCD-mediated PCD [35]. However, the enrichment analysis did not uncover any GO terms related to PCD (Supplementary Tables S6 and S7). Furthermore, the upregulation of BnaC09g20030D, with sequence similarity to a Bax inhibitor-1, might negate the effect of BnaCnng58090D by inhibiting PCD [32]. The hypersensitivity that DCD would otherwise be induced in response to infection might be prevented by the upregulation of BnaC09g20030D in Westar.

One of the DEGs upregulated uniquely in inoculated Westar is a heat-shock protein 70, which might be linked to the upregulation of Bax inhibitor-1. Qi et al. [36] noted that the overexpression of a heat-shock protein 70 inhibited PCD induced by HP. This might explain the results of this study, i.e., that the plant cell death (lesion) generally fell behind HP production and *Lm* hyphal growth in Westar. The lack of observed differences in genomic DNA degradation failed to support this hypothesis. Although fragmentation of genomic DNA can be associated with PCD, including that mimicking apoptosis (in animal cells) and that involved in normal plant developmental processes [37], the results of Ruberti et al. [38] showed complex roles of Bax inhibitor-1 in plant PCD, which is currently not well understood. Therefore, the results of indifference in genomic-DNA degradation as a proxy for PCD cannot be clearly interpreted at this point.

PCD in general, and Bax inhibitor-1 in particular, play a role in plant resistance to pathogens. For example, Babaeizad et al. [39] found that the overexpression of Bax inhibitor-1 in barley led to increased susceptibility to the biotrophic fungal pathogen *Blumeria graminis* f.sp. *hordei* (powdery mildew). This finding was consistent with the upregulation of a DEG related to Bax inhibitor-1 in Westar, corresponding to a greater biotrophic growth of the *Lm* hyphae asymptomatically in live cells beyond necrotic tissues (lesions) on cotyledons. Increased PCD is associated with the resistance to biotrophic infection and susceptibility to necrotrophic colonization by fungal pathogens. Scotton et al. [40] suggested that constitutive overexpression of Bax inhibitor-1 result in elevated resistance to necrotrophic fungal pathogens. *Lm* is considered a hemibiotroph [19], with an initial biotrophic infection, followed by extensive necrotrophic colonization of host leaf tissues.

**Figure 10:**
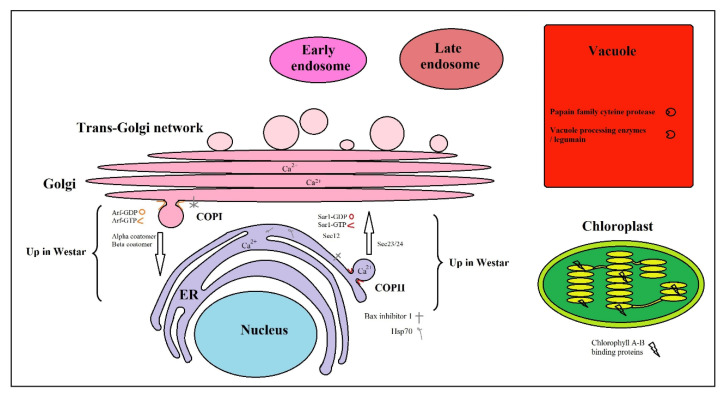
A proposed model on how some of the most highly-expressed DEGs (differentially expressed genes) might interact, potentially resulting in programed cell death. ER: Endoplasmic reticulum.

In inoculated 74-44 BL, many genes related to peptidases were more highly expressed than in inoculated Westar; these included those related to papain cysteine peptidases (BnaA01g17570D, BnaA09g52180D) [41,42] and legumain peptidase C13 (BnaA01g04000D), also known as a vacuole processing enzyme (VPE). VPEs, as suggested by the name, are located in plant vacuoles [43,44], as illustrated in Figure 10. In addition, the gene BnaC02g00130D, which putatively encodes a protease involved in the degradation of RuBisCO, was upregulated. These genes might also be involved in PCD (reviewed by Zamyatnin [45]), which causes the plant cell vacuole to rupture, releasing proteases that degrade cellular components [46]. Protease-mediated PCD is essential for plant hypersensitive responses (reviewed by Sueldo and van der Hoorn [47]), which limit infection during the biotrophic phase. These proteases merit further investigation for potential involvement in QR.

The protease inhibitor experiments were intended to verify the role for some of the peptidases in limiting *Lm* hyphal growth in inoculated 74-44 BL, which could possibly occur through a role in PCD. However, there was a lack of significant protease- or peptidase-related GO terms in the enrichment analysis (Supplementary Table S14), which would call into question the significance of proteases in the resistance. The lack of differences between inhibitor treatments, coupled with the insignificant GO terms, did not support proteases as important players in QR with 74-44 BL, despite strong upregulation of several peptidase-related genes.

Several genes related to chlorophyll A-B binding proteins (which are a source of ROS [48]) were also upregulated in inoculated 74-44 BL and thus might be involved in QR. ROS, including HP, can act as pro-PCD signals (reviewed by Galvez-Valdivieso and Mullineaux [49]). This was supported by the findings from the current study where HP production occurred beyond the lesion development and *Lm* hyphal fronts in inoculated 74-44 BL (Figure 6 and Figure 7). The HP production (ROS) appeared to result in rapid plant cell death that restricted *Lm* hyphal growth. In contrast, *Lm* hyphal growth kept in pace with the circle caused by HP beyond the lesion, showing little restriction in Westar. It appeared that ROS in 74-44 BL resulted in a near-hypersensitive reaction rapidly surrounding the *Lm* hyphae, limiting the biotrophic phase of infection beyond the lesion. Additional mechanisms aside from ROS might also play a role. BnaA03g11710D was another DEG in the photosynthetic process upregulated in inoculated 74-44 BL, which putatively encodes the thiazole biosynthetic enzyme or ribulose-1,5-bisphosphate synthetase. Thiazole is a precursor of vitamin B1 (thiamine) which can activate plant defenses [50]. Ahn et al. [51] and Boubakri et al. [52] found a relationship between vitamin B1-induced disease resistance and HP. Consistently, KEGG analysis also identified BnaA03g11710D as being involved in thiamine metabolism (Supplementary Table S14). These results showed a link between upregulation of BnaA03g11710D and increased HP production in inoculated 74-44 BL, further strengthening the case for this DEG being involved in QR. Additionally, three GO terms linked to HP were also identified (Supplementary Table S14), adding support to ROS involvement in QR.

The DEGs unique to mock-inoculated Westar and 74-44 BL provide some insight into the differences between the two cultivars, independent of *Lm* infection. For example, a WD40 repeat-containing protein and a putative C2 calcium-dependent membrane targeting protein, both of which could play a role in signaling [53,54], were upregulated in the 74-44 BL, while a gene potentially involved in intracellular signaling was upregulated in Westar (Supplementary Tables S6 and S7). These might suggest that signaling networks differ somewhat between these two cultivars, in the absence of *Lm* infection. The higher expression of certain genes putatively encoding a late embryogenesis abundance protein, a disease resistance protein and thaumatin (which could also be involved in plant disease resistance [55]) in Westar, without exposure to *Lm*, might point to inherent differences between the cultivars (Supplementary Table S13). Clearly, these putative defense-related genes, at least at the levels they are expressed in Westar, appear insufficient to prevent infection of Westar by *Lm*.

The DEGs involved in endomembrane trafficking, such as small GTPases, sec23/sec24, sec61 and WD40 repeats, can be involved in ER stress and unfolded protein responses (UPR); if not resolved, both processes can lead to PCD (reviewed by Williams et al. [56]). Vesicle trafficking between the Golgi and ER, and vice versa, affects traffic to the vacuole, where VPE-mediated PCD takes place (Figure 10). VPE is one of the proteases upregulated in inoculated 74-44 BL. UPR is triggered by improperly folded proteins in ER; if the stress to ER is severe, UPR could also induce PCD (reviewed by Cui et al. [51]). As differences were not observed in genomic DNA degradation, the UPR might only be a signaling mechanism related to QR, rather than PCD. Further research into the potential roles of UPR, endomembrane dynamics, and ROS in plant defenses is merited for a better understanding of QR against the blackleg of canola.

## 4. Materials and Methods

This manuscript includes the following five experiments on the cotyledons of Westar (susceptible) and 74-44 BL, with a level of QR against the different *Lm* races [11,12]: (1) RNA-seq and corresponding infection assessment, (2) a time-course evaluation on necrotic lesion development corresponding to *Lm* colonization post inoculation, (3) staining for HPwith DAB as an indicator for the production of ROS, (4) a protease-inhibitor study to validate the role of peptidases in resistance to *Lm*, and (5) an assessment of the level of fragmentation of genomic DNA as a proxy for PCD.

### 4.1. Fungal and Plant Material

Inoculum was prepared from *L. maculans* isolates 12CC09 carrying *AvrLm6,7* and 12CC09-GFP, grown on V8 agar for about 10 days, until pycnidia were visible. The isolate, 12CC09, was selected for this study, as it lacked *AvrLm1*, *AvrLm3*, and *AvrLmS*, which would interact with the R genes *Rlm1*, *Rlm3*, or *RlmS* in 74-44 BL [11]. Pycnidiospores were harvested in sterile water, filtered through a Falcon™ Cell Strainer (70 μm pore size), diluted to 2 × 10^7^ spores/mL, and stored at −20 °C until use. One week after planting, the cotyledons were wounded on each lobe with bent-tipped tweezers before being inoculated with 10 μl droplets of water or pycnidiospore suspension.

The isolate 12CC09-GFP was generated by transforming the isolate 12CC09 with a binary vector containing the GFP gene via *Agrobacterium*-mediated transformation. Aliquots of 1 mL *Lm* 12CC09 suspension (1 × 10^7^ spores/mL) were mixed with 1 mL of log-phase *Agrobacterium tumefaciens* Agl1 cells carrying the GFP vector. The cell mixture was pelleted by centrifugation, resuspended with the liquid AB-MES medium [57], and spread on sterile 0.45 μm nitrocellulose membranes (GE Healthcare, Ottawa, ON, Canada) overlaid on a solid AB-MES medium. After 3-day incubation in darkness at room temperature, the membranes were cut into 0.5-cm strips, placed on a selection medium (solid V8 containing 50 μg/mL hygromycin and 50 μg/mL carbenicillium), and incubated at 24 °C for 7–10 days. Pycnidial ooze from single colonies was transferred individually to the selection medium and the resulting cultures (potential transformants) were assessed for GFP expression with a Zeiss Stereo-Lumar epifluorescence microscope. The isolate 12CC09-GFP expressed strong and consistent GFP signal in a pure culture and in canola leaf tissues (Figure 1C,D).

74-44 BL is DEKALB^®^ hybrid with multi-genic *Lm* resistance and R genes *Rlm1*, *Rlm3*, and *RlmS* (Saskatchewan Seed Guide, 2019). The differential reaction of the 74-44 BL cotyledons to *Lm* isolates with and without *AvrLm1* and *AvrLm3* (Supplementary Figure S1) confirmed the presence of *Rlm1*/*LepR3* and *Rlm3*. 74-44 BL also carries a level of QR against multiple *Lm* races in cotyledons [12], with lower lesion scores [32], more restricted *Lm* colonization [12], as well as QR displayed in adult plants in the absence of major-gene interactions [32].

Plants were grown in Sunshine #3 soil-less mix (Sun Gro Horticulture Canada Ltd., Vancouver, BC, Canada) to which 12.5 g L^−1^ Osmocote Plus 16-9-12 (N-P-K; Scotts Miracle-Gro Canada, Mississauga, ON, Canada) was added. For all experiments, except those involving the time series that did not involve DAB staining, canola plants were grown in 72-well flats. The flats were placed in a growth chamber set to 22 °C and 16 °C, during the 16 h of light (approximately 280–575 μmol m^−2^ s^−1^) and 8 h of darkness, respectively. Plants intended for the time-series microscopic examination were grown either as described above or in the greenhouse, in 10 cm square pots, exposed to a mix of natural and fluorescent (430W Philips high pressure sodium lamps) light, and inoculated with water, 12CC09, or 12CC09-GFP. Isolate 12CC09 was included as a control to determine if the fluorescence observed could be attributed to GFP.

Plants were divided into *Lm*-inoculated and mock-inoculated. Within each inoculation treatment, plants were split between cultivars. The RNA-seq and time-series microscopy experiments were repeated 3 times, as were the experiments involving staining for HP (ROS) with DAB. The protease inhibitor experiments were carried out five times.

For the RNA-seq experiments, within each replicate, there were six seedlings per treatment (Westar or 74-44 BL, mock, or 12CC09-GFP-inoculated), divided at random into two blocks of three plants. At 7 dpi, the cotyledon samples were taken from three of these seedlings and pooled as a replicate for RNA extraction and subsequent RNA-seq. The remaining three seedlings were kept until 14 dpi and rated for infection severity on the 0–9 scale [58,59]. The experiment was conducted three times, resulting in three independent replicates per treatment for the RNA-seq experiment.

### 4.2. RNA Extraction, Library Preparation, and Sequencing

Samples, measuring 5–10 mm × 5–10 mm, were collected from the area adjacent to and containing the lesion on each lobe of the cotyledons at 7 dpi (Figure 1A). Samples were flash frozen in liquid nitrogen and stored at −80 °C until RNA extraction. Only one of the inoculated lobes was taken from each plant and pooled for the replicate (3 plants). A total of 12 pooled samples from four treatments, three replicates per treatment, were used for RNA extraction.

Cotyledon tissue was ground in liquid nitrogen by vortexing in 50 mL Nalgene Oak Ridge tubes containing two metal beads. RNA was extracted from 40–50 mg of the ground and frozen tissue using the QIAGEN RNeasy Plant Mini Kit on a QIAcube with a DNase I on-column digestion. The concentration and integrity of the resulting RNA was assessed via Nanodrop and Experion (Bio-Rad Canada, Mississauga, ON, Canada) automated electrophoresis, respectively.

Sequencing libraries were prepared using a Illumina^®^ TruSeq™ RNA Sample Preparation Kit. Each treatment consisted of three biological replicates, with each including samples pooled from three plants. A library was prepared for each replicate, with a total of 12 libraries from the four treatments. These libraries were sequenced on the Illumina HiSeq 2500 using one lane of V4 PE 125 bp at the Genome Quebec Innovation Center (McGill University, Montreal, QC, Canada).

### 4.3. RNA-Seq Data Analysis

Adapter sequences were removed with Trimmomatic (version 0.32) [60]. Subsequently, both paired and unpaired reads were aligned to the *B. napus* and *Lm* reference genomes ([61] and [62], respectively) via STAR (version 2.4.2a) [63]. Gene annotations for *Lm* and *B. napus* used the reference genomes from the same sources. Unpaired reads were retained to reduce the risk of introducing bias that might result from discarding these potentially shorter reads. As all samples were treated in the same manner, comparisons between treatments are still valid. Next, the gene models were defined using the GenomicFeatures package in R, and the reads were counted using the R package GenomicAlignments [64]. Differential expression analysis was conducted in R (version 3.3.1 or 3.3.2) using the DESeq2 package for non-normalized read counts (as a count matrix within an assay of a RangedSummarizedExperiment) as required by the package [65]. DESeq2 normalizes samples based on the library size. Due to the importance of the relative changes in expression, statistical significance of these changes were computed between treatments and the overall gene expression (basemean); genes were considered differentially expressed if they had a log-base-2 (log_2_) fold of change in expression > 2 or < −2, we well as an adjusted *p* value ≤ 0.05. Differentially expressed genes (DEGs) were scored based on expression (basemean), adjusted *p* value (adjp) and log_2_-fold changes in expression (basemean):

(1) $$Score=Baseman\times \left[\log_{2}\left(fold\;change\;in\;expression\right)\right]\times \left[-\log_{2}\left(adjp\right)\right]$$

Venn diagrams (Figure 4) were used to identify DEGs that were unique to each combination of contrasting treatments—inoculated Westar versus inoculated 74-44 BL, mock-inoculated Westar versus mock-inoculated 74-44 BL, mock versus *Lm*-inoculated Westar, and mock versus *Lm*-inoculated 74-44 BL. DEGs were also subdivided into those with higher expression in the former of the two treatments, being contrasted (positive, Figure 4A) and those upregulated in the latter of the two contrasted treatments (negative, Figure 4B).

KEGG and gene ontology (GO) annotation for DEGs in different groups were extracted from an Ensemble Plant database, using the R biomaRt package. GO enrichment was performed using the enricher function of the R clusterProfiler package. GO annotation and enrichment was also performed using the Blast2Go-pro suite [66]. In this method of GO enrichment, all *B. napus* genes were searched against the non-redundant protein database from the National Center for Biotechnology Information using BLASTX algorithm with an E-value threshold of 1e^−5^. All BLAST hits were mapped onto the GO database to retrieve the terms associated with each hit. Subsequently, all *B. napus* genes were searched against the InterPro database and annotated by merging the search results from both Blast2GO and InterPro. All expressed *B. napus* genes were used as a background for the enrichment if they were annotated in GO. The *p* value and false discovery rate (FDR) cut-off were both set at 0.05.

Volcano plots were performed using the R plot function, based on the results of the DEG analysis, using the R DESeq2 package. All expressed genes were clustered to plot a heatmap using the R pheatmap package, based on gene expression patterns. Z-score transformation was applied prior to heatmap plotting for normalization.

### 4.4. Colorimetric Detection of Hydrogen Peroxide

The area staining for the ROS HP in *B. napus* cotyledons infected by *Lm* isolate 12CC09-GFP was measured initially at 7 dpi. Images were collected with a Zeiss Stereo-Lumar epifluorescence microscope, equipped with a NeoLumar S 0.8× objective and an Axiocam 512 camera. Light was provided by a KL-2500 LCD (bright field) or a HBO100 mercury (fluorescent images) bulb. Subsequently, the detached cotyledons were placed in a solution of DAB at room temperature. After 40 (first two experiments) or 90 (third experiment) min, the cotyledons were vacuum infiltrated with DAB for approximately 2 to 3 h. Samples were then boiled in 95% ethanol for approximately 10 to 20 min at 70 °C, to remove the chlorophyll, making the DAB staining more visible. The cotyledons were stored in 95% ethanol, prior to measurement of the area stained brown for HP under a dissecting microscope.

The lesion size, the area colonized by GFP-tagged *Lm* hyphae, and the area of DAB staining was measured with the aid of ZEN 2 pro or ZEN 2.3 lite (blue edition, © Carl Zeiss Microscopy GmbH, 2011) software, which automatically accounted for magnification.

### 4.5. Time-Series Infection in Cotyledons

In an initial experiment, the inoculated cotyledons were detached from the plant for microscopic examination at 3, 7, 10, and 14 dpi. The experiment was repeated with narrower time intervals of sampling, in order to obtain more detailed time-series data at 3, 5, 7, 9, and 11 dpi. The later experiments also included colorimetric staining for HP. This was done to compliment and confirm colorimetric detection of HP at 7 dpi, as described above. In each experiment, destructive sampling was used at each time point. As the lesion area and the area colonized by the *Lm* hyphae at 3 dpi were frequently zero, data from this time-point was not included in the analyses.

Bright field images of the top surface of each cotyledon were used to measure the area of the lesion (mm^2^), the distance from the inoculation point to the most distant edge of the lesion (mm), and the area stained HP (mm^2^). The area colonized by hyphae (mm^2^) and the distance from the edge of inoculation wound to the furthest hyphal tip (mm) (first set of time-series experiments only) were quantified using fluorescent images. The Zen Active Contour or Polygon Contour and Length tools were used to collect the area and distance data, respectively.

### 4.6. Assessment of Genomic DNA Degradation as a Marker of Programmed Cell Death

Samples of canola cotyledons were collected as described for the RNA extraction. The samples were freeze dried, and ground to a fine powder in 2 mL tubes, with one 3 mm tungsten carbide bead per tube in a TissueLyser (Qiagen), at room temperature for 5 min at 25 hertz. Genomic DNA was extracted using the QIAGEN DNeasy Plant Mini Kit, according to the manufacturer’s instructions. Extracted DNA was diluted to 50 ng/μL. The integrity of the resulting DNA was assessed using Experion DNA 12K analysis kit (Bio-Rad Canada) on an Experion automated electrophoresis system, according to the manufacturer’s instructions.

### 4.7. Statistical Analysis

Statistical analyses were done using SAS (version 9.3). Data were assessed for homogeneity of variance and normality, respectively, using Bartlett’s Test and the Shapiro-Wilk Test. Data from mock-inoculated plants, which consisted exclusively of zeros, were excluded from statistical analysis.

For ratings of infection severity in parallel with RNA-seq, a randomized complete block design (RCBD) was used, with 3 replicates per treatment and 3 subsamples (plants) per replicate (Figure 1B). These data were pooled from all plants in a given experiment and y = log_10_(x + 10) transformed. Means were compared via a *t*-test, using Proc GLM. For the time-series experiments that did not include HP assessment (Figure 1E), data were y = log_10_(x + 10) transformation. Each parameter (lesion area, area colonized by *Lm* hyphae, distance to the edge of lesion, or to furthest hyphal tips from the inoculation site) and time-point (7, 10 or 14 dpi) were analyzed individually using *t*-test to compare the means between cultivars. As much of these data did not meet all requirements for ANOVA, they were analyzed using the Wilcoxon two-sample (for cultivar) or Kruskal–Wallis multi-sample (for comparisons between parameters) test, using the Proc NPAR1WAY. When significant differences were found for a given factor (cultivar or parameter), the means of treatments were separated using Tukey’s adjusted multiple comparison. For the protease inhibitor experiment (Figure 9), a two-factor (cultivar and protease inhibitor) analysis was performed, and Tukey’s adjustment was also used for mean comparison.

## 5. Conclusions

QR observed in 74-44 BL cotyledons appeared to result from restricted *Lm* colonization due, at least in part, to ROS, which might trigger a rapid PCD via signal transduction and endomembrane vesicle trafficking. The upregulation of genes related to a putative Bax inhibitor-1 might reduce the PCD response in the susceptible Westar. The restriction of cotyledon colonization by *Lm* could be of significant benefit to blackleg resistance as it might limit or prevent the pathogen from spreading into the stem, where the most damaging form of the disease takes place. This study served as a prelude to further research into mechanisms involved in QR against blackleg. Such work, in conjunction with studies of more resistant canola cultivars, might facilitate judicious deployment of QR traits for improved blackleg management.

## Supplementary Materials

The following are available online at https://www.mdpi.com/2223-7747/9/7/864/s1, Figure S1: Differential reaction of Westar and 74-44 BL cotyledons to inoculation with *L. maculans* isolates carrying *AvrLm1* (S7) or *AvrLm3* (19.4.24), or lacking *AvrLm1* and *AvrLm3* (12CC-09), Table S1: RNA-seq read mapping, Table S2. Genes of *Leptosphaeria maculans* that are more highly expressed in inoculated 74-44 BL than in inoculated Westar, Table S3. Genes of *Leptosphaeria maculans* that are more highly expressed in inoculated Westar than in inoculated 74-44 BL, Table S4. Genes of *Brassica napus* that are more highly expressed in *Lm*-inoculated cotyledons of 74-44 BL than those of Westar (score ≤ −2 × 10^4^), Table S5. Gene Ontology (GO) term enrichment of differentially expressed genes (DEGs) in *Brassica napus* that are more highly expressed in *Lm*-inoculated 74-44 BL than in inoculated Westar, Table S6. KEGG annotation of differentially expressed genes (DEGs) in *Brassica napus* that are more highly expressed in *Lm*-inoculated 74-44 BL than in inoculated Westar (scores > 1.0 × 10^3^), Table S7. Genes of *Brassica napus* that are more highly expressed in *Lm*-inoculated cotyledons of Westar than in those of 74-44 BL (scores > 2 × 10^4^), Table S8. GO term enrichment of DEGs in *Brassica napus* that are more highly expressed in *Lm*-inoculated Westar than in inoculated 74-44 BL, Table S9. KEGG annotation of differentially expressed genes (DEGs) in *Brassica napus* that are more highly expressed in *Lm*-inoculated Westar than in inoculated 74-44 BL (scores > 1.0 × 10^3^), Table S10. Genes of *Brassica napus* that are more highly expressed in mock-inoculated cotyledons of 74-44 BL than in those of mock Westar (scores < 2 × 10^4^), Table S11. GO term enrichment of DEGs in *Brassica napus* that are more highly expressed in mock-inoculated 74-44 BL than in mock Westar, Table S12. KEGG annotation of differentially expressed genes (DEGs) in *Brassica napus* that are more highly expressed in mock-inoculated 74-44 BL than in mock-inoculated Westar (scores > 1.0 × 10^3^), Table S13. Genes of *Brassica napus* that are more highly expressed in mock-inoculated cotyledons of Westar than in those of 74-44 BL (scores > 8.6 × 10^3^), Table S14. GO term enrichment of DEGs in *Brassica napus* that are more highly expressed in mock-inoculated Westar than in mock 74-44 BL, Table S15. KEGG annotation of differentially expressed genes (DEGs) in *Brassica napus* that are more highly expressed in mock-inoculated Westar than in mock 74-44 BL (scores >1.0 × 10^3^).

## References

[1] Fitt B.D.L., Brun H., Barbetti M.J., Rimmer S.R. (2006). World-Wide Importance of Phoma Stem Canker (Leptosphaeria maculans and L. biglobosa) on Oilseed Rape (*Brassica napus*). Eur. J. Plant Pathol..

[2] Delourme R., Chevre A.-M., Brun H., Rouxel T., Balesdent M.H., Dias J.S., Salisbury P., Renard M., Rimmer S.R. (2006). Major Gene and Polygenic Resistance to Leptosphaeria maculans in Oilseed Rape (*Brassica napus*). Eur. J. Plant Pathol..

[3] Cowger C., Brown J.K.M. (2019). Durability of Quantitative Resistance in Crops: Greater Than We Know?. Annu. Rev. Phytopathol..

[4] Larkan N., Lydiate D.J., Parkin I.A.P., Nelson M.N., Epp D.J., Rimmer S.R., Cowling W.A., Borhan H. (2012). The *Brassica napus* blackleg resistance gene lepr3 encodes a receptor-like protein triggered by the Leptosphaeria maculans effector AVRLM1. New Phytol..

[5] Yu F., Lydiate D.J., Rimmer S.R. (2005). Identification of two novel genes for blackleg resistance in *Brassica napus*. Theor. Appl. Genet..

[6] Yu F., Gugel R.K., Kutcher H.R., Peng G., Rimmer S.R. (2012). Identification and mapping of a novel blackleg resistance locus *LepR4* in the progenies from *Brassica napus* × *B. rapa* subsp. Sylvestris. Theor. Appl. Genet..

[7] Raman R., Taylor B., Lindbeck K., Coombes N., Barbulescu D., Salisbury P., Raman H. (2012). Molecular and Molecular mapping and validation of Rlm1 gene for resistance to *Leptosphaeria maculans* in canola (*Brassica napus L.*). Crop. Pasture Sci..

[8] Larkan N., Lydiate D.J., Yu F., Rimmer S.R., Borhan H. (2014). Co-localisation of the blackleg resistance genes Rlm2 and LepR3 on *Brassica napus* chromosome A10. BMC Plant Biol..

[9] Parlange F., Daverdin G., Fudal I., Kuhn M.-L., Balesdent M.-H., Blaise F., Grezes-Besset B., Rouxel T. (2009). *Leptosphaeria maculans* avirulence gene AvrLm4-7 confers a dual recognition specificity by theRlm4andRlm7resistance genes of oilseed rape, and circumventsRlm4-mediated recognition through a single amino acid change. Mol. Microbiol..

[10] Kumar V., Paillard S., Fopa-Fomeju B., Falentin C., Deniot G., Baron C., Vallée P., Manzanares-Dauleux M.J., Delourme R. (2018). Multi-year linkage and association mapping confirm the high number of genomic regions involved in oilseed rape quantitative resistance to blackleg. Theor. Appl. Genet..

[11] Raman H., Raman R., Diffey S., Qiu Y., McVittie B., Barbulescu D.M., Salisbury P.A., Marcroft S., Delourme R. (2018). Stable Quantitative Resistance Loci to Blackleg Disease in Canola (*Brassica napus* L.) Over Continents. Front. Plant Sci..

[12] Soomro W.M. (2016). Characterizing Avr Genes of Leptosphaeria maculans and Resistance Responses among Commercial Canola Cultivars in Western.

[13] Poland J., Balint-Kurti P., Wisser R.J., Pratt R.C., Nelson R.J. (2009). Shades of gray: The world of quantitative disease resistance. Trends Plant Sci..

[14] Larkan N., Raman H., Lydiate D.J., Robinson S.J., Yu F., Barbulescu D.M., Rosy R., Luckett D.J., Burton W., Wratten N. (2016). Multi-environment QTL studies suggest a role for cysteine-rich protein kinase genes in quantitative resistance to blackleg disease in *Brassica napus*. BMC Plant Biol..

[15] Raman H., Raman R., Coombes N., Song J., Diffey S., Kilian A., Lindbeck K., Barbulescu D.M., Batley J., Edwards D. (2016). Genome-wide Association Study Identifies New Loci for Resistance to *Leptosphaeria maculans* in Canola. Front. Plant Sci..

[16] Fomeju B.F., Falentin C., Lassalle G., Manzanares-Dauleux M.J., Delourme R. (2014). Homoeologous duplicated regions are involved in quantitative resistance of *Brassica napus* to stem canker. BMC Genom..

[17] Jestin C., Lode M., Vallee P., Domin C., Falentin C., Horvais R., Coedel S., Manzanares-Dauleux M.J., Delourme R. (2010). Association mapping of quantitative resistance for *Leptosphaeria maculans* in oilseed rape (*Brassica napus* L.). Mol. Breed..

[18] Haddadi P., Ma L., Wang H., Borhan M.H. (2015). Genome-wide transcriptome analyses provides insights into the lifestyle transition and effector repertoire of *Leptosphaeria maculans* during colonization of *Brassica napus* seedlings. Mol. Plant Pathol..

[19] Sonah H., Zhang X., Deshmukh R., Borhan M.H., Fernando W.G.D., Bélanger R.R. (2016). Comparative Transcriptomic Analysis of Virulence Factors in *Leptosphaeria maculans* during Compatible and Incompatible Interactions with Canola. Front. Plant Sci..

[20] Becker M.G., Zhang X., Walker P.L., Wan J.C., Millar J.L., Khan D., Granger M.J., Cavers J.D., Chan A.C., Fernando D.W. (2017). Transcriptome analysis of the *Brassica napus-Leptosphaeria maculans* pathosystem identifies receptor, signaling and structural genes underlying plant resistance. Plant J..

[21] Zhou T., Xu W., Hirani A.H., Liu Z., Tuan P.A., Ayele B.T., Daayf F., McVetty P.B.E., Duncan R.W., Li G. (2019). Transcriptional Insight Into *Brassica napus* Resistance Genes LepR3 and Rlm2-Mediated Defense Response Against the *Leptosphaeria maculans* Infection. Front. Plant Sci..

[22] Hao Y., Wang T., Wang K., Wang X., Fu Y., Huang L., Kang Z. (2016). Transcriptome Analysis Provides Insights into the Mechanisms Underlying Wheat Plant Resistance to Stripe Rust at the Adult Plant Stage. PLoS ONE.

[23] Joshi R.K., Megha S., Rahman M.H., Basu U., Kav N.N. (2016). A global study of transcriptome dynamics in canola (*Brassica napus* L.) responsive to Sclerotinia sclerotiorum infection using RNA-Seq. Gene.

[24] Huang Y.-J., Jestin C., Welham S.J., King G.J., Manzanares-Dauleux M.J., Fitt B.D.L., Delourme R. (2015). Identification of environmentally stable QTL for resistance against *Leptosphaeria maculans* in oilseed rape (*Brassica napus*). Theor. Appl. Genet..

[25] Huang Y.-J., Qi A., King G.J., Fitt B.D.L. (2014). Assessing Quantitative Resistance against *Leptosphaeria maculans* (Phoma Stem Canker) in *Brassica napus* (Oilseed Rape) in Young Plants. PLoS ONE.

[26] Wendenbaum S., Demange P., Dell A., Meyer J., Abdallah M. (1983). The structure of pyoverdine Pa, the siderophore of Pseudomonas aeruginosa. Tetrahedron Lett..

[27] Zwiers L.-H., Roohparvar R., De Waard M.A. (2007). MgAtr7, a new type of ABC transporter from Mycosphaerella graminicola involved in iron homeostasis. Fungal Genet. Boil..

[28] Kim Y., Park S.-Y., Kim D., Choi J., Lee Y.-H., Lee J.-H., Choi W. (2013). Genome-scale analysis of ABC transporter genes and characterization of the ABCC type transporter genes in Magnaporthe oryzae. Genomics.

[29] Yin Y., Wang Z., Cheng D., Chen X., Chen Y., Ma Z. (2018). The ATP-binding protein FgArb1 is essential for penetration, infectious and normal growth ofFusarium graminearum. New Phytol..

[30] Yorimitsu T., Sato K., Takeuchi M. (2014). Molecular mechanisms of Sar/Arf GTPases in vesicular trafficking in yeast and plants. Front. Plant Sci..

[31] Bioinformatics & Evolutionary Genomics. http://bioinformatics.psb.ugent.be/webtools/Venn/.

[32] Hubbard M., Peng G. (2018). Quantitative resistance against an isolate of *Leptosphaeria maculans* (blackleg) in selected Canadian canola cultivars remains effective under increased temperatures. Plant Pathol..

[33] Huang Y.-J., Paillard S., Kumar V., King G.J., Fitt B.D.L., Delourme R. (2019). Oilseed rape (*Brassica napus*) resistance to growth of *Leptosphaeria maculans* in leaves of young plants contributes to quantitative resistance in stems of adult plants. PLoS ONE.

[34] Tenhaken R., Doerks T., Bork P. (2005). DCD—A novel plant specific domain in proteins involved in development and programmed cell death. BMC Bioinform..

[35] Reis P.A.B., Carpinetti P.A., Freitas P.P., Santos E.G., Camargos L.F., De Oliveira I.H.T., Silva J.C.F., Carvalho H.H., Dal-Bianco M., Soares-Ramos J.R. (2016). Functional and regulatory conservation of the soybean ER stress-induced DCD/NRP-mediated cell death signaling in plants. BMC Plant Biol..

[36] Qi Y., Wang H., Zou Y., Liu C., Liu Y., Wang Y., Zhang W. (2010). Over-expression of mitochondrial heat shock protein 70 suppresses programmed cell death in rice. FEBS Lett..

[37] Hoeberichts F.A., De Jong A.J., Woltering E.J. (2005). Apoptotic-like cell death marks the early stages of gypsophila (Gypsophila paniculata) petal senescence. Postharvest Boil. Technol..

[38] Ruberti C., Lai Y., Brandizzi F. (2017). Recovery from temporary endoplasmic reticulum stress in plants relies on the tissue-specific and largely independent roles of bZIP28 and bZIP60, as well as an antagonizing function of BAX-Inhibitor 1 upon the pro-adaptive signaling mediated by bZIP28. Plant J..

[39] Babaeizad V., Imani J., Kogel K.-H., Eichmann R., Huckelhoven R. (2008). Over-expression of the cell death regulator BAX inhibitor-1 in barley confers reduced or enhanced susceptibility to distinct fungal pathogens. Theor. Appl. Genet..

[40] Scotton D.C., Azevedo M.D.S., Sestari I., Da Silva J.S., Souza L.A., Peres L.E.P., Leal G.A., Figueira A. (2016). Expression of the Theobroma cacao Bax-inhibitor-1 gene in tomato reduces infection by the hemibiotrophic pathogen Moniliophthora perniciosa. Mol. Plant Pathol..

[41] Diaz-Mendoza M., Velasco-Arroyo B., González-Melendi P., Martinez M., Diaz I. (2014). C1A cysteine protease–cystatin interactions in leaf senescence. J. Exp. Bot..

[42] Okamoto T., Shimada T., Hara-Nishimura I., Nishimura M., Minamikawa T. (2003). C-Terminal KDEL Sequence of A KDEL-Tailed Cysteine Proteinase (Sulfhydryl-Endopeptidase) Is Involved in Formation of KDEL Vesicle and in Efficient Vacuolar Transport of Sulfhydryl-Endopeptidase1. Plant Physiol..

[43] Hara-Nishimura I., Inoue K., Nishimura M. (1991). A unique vacuolar processing enzyme responsible for conversion of several proprotein precursors into the mature forms. FEBS Lett..

[44] Hara-Nishimura I., Nishimura M. (1987). Proglobulin Processing Enzyme in Vacuoles Isolated from Developing Pumpkin Cotyledons. Plant Physiol..

[45] Zamyatnin A.A. (2015). Plant Proteases Involved in Regulated Cell Death. Biochemistry (Moscow).

[46] Zheng Y., Zhang H., Deng X., Liu J., Chen H. (2017). The relationship between vacuolation and initiation of PCD in rice (Oryza sativa) aleurone cells. Sci. Rep..

[47] Sueldo D.J., Van Der Hoorn R.A.L. (2017). Plant life needs cell death, but does plant cell death need Cys proteases?. FEBS J..

[48] Xu Y.-H., Liu R., Yan L., Liu Z.-Q., Jiang S.-C., Shen Y.-Y., Wang X.-F., Zhang D.-P. (2011). Light-harvesting chlorophyll a/b-binding proteins are required for stomatal response to abscisic acid in Arabidopsis. J. Exp. Bot..

[49] Gálvez-Valdivieso G., Mullineaux P.M. (2010). The role of reactive oxygen species in signalling from chloroplasts to the nucleus. Physiol. Plant..

[50] Ahn I.-P., Kim S., Lee Y.-H. (2005). Vitamin B1 Functions as an Activator of Plant Disease Resistance1. Plant Physiol..

[51] Ahn I.-P., Kim S., Lee Y.-H., Suh S.-C. (2006). Vitamin B1-Induced Priming Is Dependent on Hydrogen Peroxide and the NPR1 Gene in Arabidopsis. Plant Physiol..

[52] Boubakri H., Wahab M.A., Chong J., Bertsch C., Mliki A., Soustre-Gacougnolle I. (2012). Thiamine induced resistance to *plasmopara viticola* in grapevine and elicited host–defense responses, including HR like-cell death. Plant Physiol. Biochem..

[53] Jain B.P., Pandey S. (2018). WD40 Repeat Proteins: Signalling Scaffold with Diverse Functions. Protein J..

[54] Nalefski E.A., Wisner M.A., Chen J.Z., Sprang S.R., Fukuda M., Mikoshiba K., Falke J.J. (2001). C2 Domains from Different Ca2+ Signaling Pathways Display Functional and, Mechanistic Diversity. Biochemistry.

[55] Andersen E.J., Ali S., Byamukama E., Yen Y., Nepal M.P. (2018). Disease Resistance Mechanisms in Plants. Genes.

[56] Williams B., Verchot J., Dickman M. (2014). When supply does not meet demand-ER stress and plant programmed cell death. Front. Plant Sci..

[57] Hwang H.-H., Wang M.-H., Lee Y.-L., Tsai Y., Li Y.-H., Yang F.-J., Liao Y.-C., Lin S., Lai E.-M. (2010). Agrobacterium-produced and exogenous cytokinin-modulated Agrobacterium-mediated plant transformation. Mol. Plant Pathol..

[58] Kutcher H., Balesdent M.H., Rimmer S.R., Rouxel T., Chevre A.-M., Delourme R., Brun H. (2010). Frequency of avirulence genes in *Leptosphaeria maculans* in western Canada. Can. J. Plant Pathol..

[59] Koch E., Badawy H.M.A., Hoppe H.H. (1989). Differences Between Aggressive and Non-Aggressive Single Spore Lines of *Leptosphaeria maculans* in Cultural Characteristics and Phytotoxin Production. J. Phytopathol..

[60] Bolger A.M., Lohse M., Usadel B. (2014). Trimmomatic: A flexible trimmer for Illumina sequence data. Bioinformatics.

[61] Genoscope. http://www.genoscope.cns.fr/brassicanapus/data/.

[62] Joint Genome Institute, Genome Portal. http://genome.jgi.doe.gov/.

[63] Dobin A., Davis C.A., Schlesinger F., Drenkow J., Zaleski C., Jha S., Batut P., Chaisson M., Gingeras T.R. (2012). STAR: Ultrafast universal RNA-seq aligner. Bioinformatics.

[64] Lawrence M., Huber W., Pagès H., Aboyoun P., Carlson M., Gentleman R., Morgan M., Carey V.J. (2013). Software for Computing and Annotating Genomic Ranges. PLoS Comput. Biol..

[65] Love M.I., Huber W., Anders S. (2014). Moderated estimation of fold change and dispersion for RNA-seq data with DESeq2. Genome Biol..

[66] Conesa A., Götz S. (2007). Blast2GO: A Comprehensive Suite for Functional Analysis in Plant Genomics. Int. J. Plant Genom..

