# Supplementary material for: Exploring Mechanisms of Quantitative Resistance to Leptosphaeria maculans (Blackleg) in the Cotyledons of Canola (Brassica napus) Based on Transcriptomic and Microscopic Analyses
Source: Plants (Basel). 2020 Jul 8;9(7):864. doi: 10.3390/plants9070864 (PMC7411684; doi:10.3390/plants9070864)
Supplement: Supplementary file 1 [file plants-09-00864-s001.pdf]

plants MDPI

Supplementary Information

A supplementary figure and 15 supplementary tables in this section contain details or data supplemental to the main figures and tables. They are crucial to understanding and reproducing the research shown, but explanation of the information may disrupt the flow of the main text.

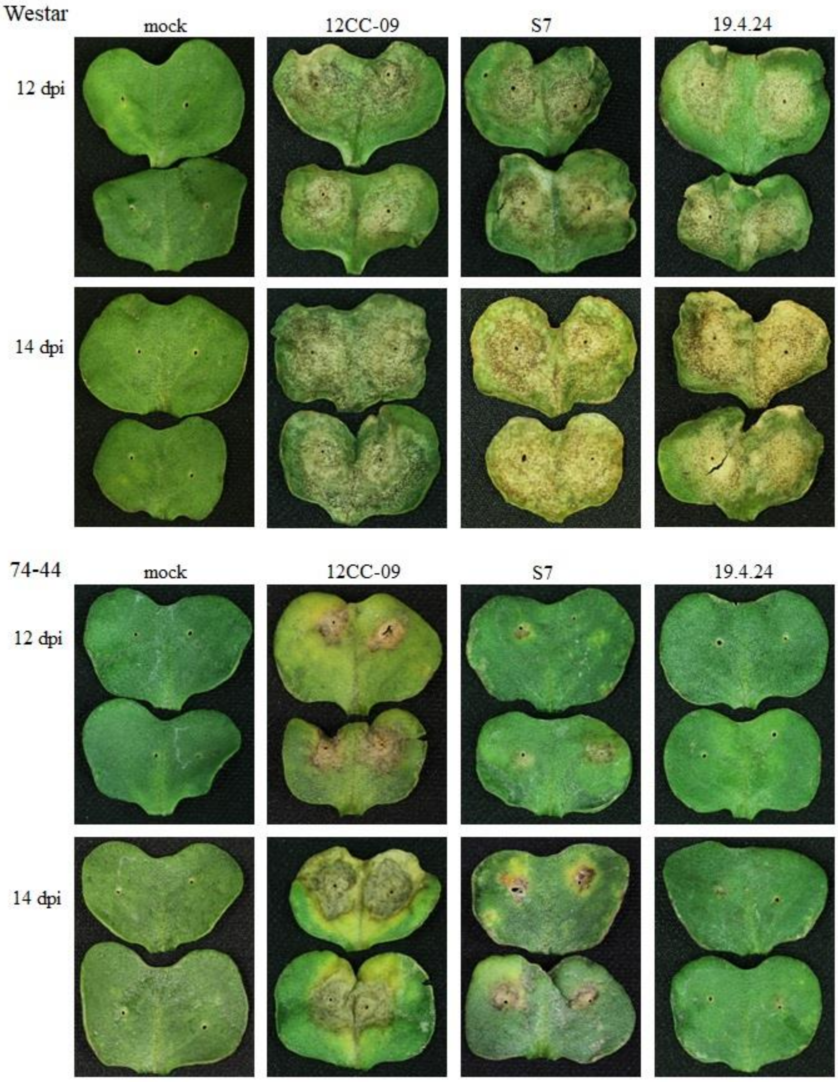

Supplementary Figure S1. Differential reaction of Westar and 74-44 BL cotyledons to inoculation with *L. maculans* isolates carrying *AvrLm1* (S7) or *AvrLm3* (19.4.24), or lacking *AvrLm1* and *AvrLm3* (12CC-09). These photos are used to illustrate the quantitative resistance (without the involvement of specific R genes) in the cotyledons of 74-44 BL; they were not part of disease severity assessment associated with any of the experiments reported in Results. The time-points (dpi) were chosen for optimal symptom expression, but might differ from those used in the reported experiments.

Supplementary Table S1. RNA-seq read mapping.

Sample Name	# Reads (pairs)	# Bases	Avg. Quality	# Reads (paired and unpaired) aligned to *B. napus* genome
74-44_Lm_Rep1	15,808,008	3,952,002,000	36	28,055,136
74-44_Lm_Rep2	17,768,395	4,442,098,750	36	31,452,049

74-44_Lm_Rep3	15,476,667	3,869,166,750	36	25,210,609
74-44_Mock_Rep1	14,640,614	3,660,153,500	36	31,043,146
74-44_Mock_Rep2	14,746,856	3,686,714,000	36	31,050,813
74-44_Mock_Rep3	17,288,454	4,322,113,500	36	36,952,881
Westar_Lm_Rep1	14,387,107	3,596,776,750	36	21,213,096
Westar_Lm_Rep2	16,540,959	4,135,239,750	36	21,392,969
Westar_Lm_Rep3	15,028,864	3,757,216,000	36	21,412,729
Westar_Mock_Rep1	16,913,431	4,228,357,750	36	37,019,069
Westar_Mock_Rep2	14,136,220	3,534,055,000	36	30,038,224
Westar_Mock_Rep3	15,741,796	3,935,449,000	36	33,496,499

Supplementary Table S2. Genes of *Leptosphaeria maculans* that are more highly expressed in inoculated 74-44 BL than in inoculated Westar.

Locus Tag	Expression	log2 fold change	Adjusted *p*-value	Score	Product	InterPro IDs	Description
LEMA_P093320.1	40.32	-2.10	8.28E-04	-2.61E+02	similar to short chain dehydrogenase/reductase family oxidoreductase	IPR002347<br>IPR016040	Short-chain dehydrogenase /reductase SDR<br>NAD(P)-binding domain
LEMA_P012380.1	12.05	-2.04	6.76E-04	-7.81E+01	hypothetical protein	IPR007817	Pyoverdine biosynthesis
LEMA_P055320.1	3.96	-2.13	1.54E-04	-3.22E+01	hypothetical protein	none	

Table S3. Genes of *Leptosphaeria maculans* that are more highly expressed in inoculated Westar than in inoculated 74-44 BL.

Locus Tag	Expression	log2 Fold Change	Adjusted *p*-value	Score	Product	InterPro IDs	Description
LEMA_P111660.1	30.84	2.11	3.36E-03	1.61E+02	predicted protein	none	
LEMA_P098600.1	7.34	2.24	1.16E-09	1.47E+02	similar to 1,3,8-naphthalenetriol reductase	IPR002347<br>IPR016040<br>IPR020904	Short-chain dehydrogenase/reductase SDR<br>NAD(P)-binding domain<br>Short-chain dehydrogenase/reductase, conserved site
LEMA_P123340.1	16.62	2.63	2.79E-03	1.12E+02	similar to amine oxidase	IPR023753	FAD/NAD(P)-binding domain
LEMA_P081810.1	12.41	2.68	1.21E-02	6.36E+01	similar to nonribosomal	IPR000873<br>IPR001242<br>IPR004045	AMP-dependent synthetase/ligase<br>Condensation domain<br>Glutathione S-transferase, N-terminal

					peptide synthase GliP	IPR004046<br>IPR006162<br>IPR009081<br>IPR010071<br>IPR010987<br>IPR012336<br>IPR020845	Glutathione S-transferase, C-terminal<br>Phosphopantetheine attachment site<br>Phosphopantetheine binding ACP domain<br>Amino acid adenylation domain<br>Glutathione S-transferase, C-terminal-like<br>Thioredoxin-like fold<br>AMP-binding, conserved site
LEMA_P07291 0.1	6.66	2.02	1.96E-03	3.65E+01	hypothetical protein	IPR023210	NADP-dependent oxidoreductase domain
LEMA_P10011 0.1	2.84	3.30	6.76E-04	2.97E+01	hypothetical protein	IPR001209	Ribosomal protein S14
LEMA_P10308 0.1	7.17	2.63	3.49E-02	2.75E+01	hypothetical protein	IPR008440<br>IPR018871	Agglutinin-like protein repeat<br>GLEYA adhesin domain
LEMA_P03204 0.1	3.21	2.39	3.32E-04	2.67E+01	hypothetical protein	IPR009078	Ferritin-like superfamily (IPR009078)
LEMA_P08173 0.1	4.72	2.31	4.36E-02	1.49E+01	similar to Methyltransferase type 11		
LEMA_P08180 0.1	3.09	3.13	4.36E-02	1.32E+01	similar to cytochrome P450	IPR001128<br>IPR002401<br>IPR017972	Cytochrome P450<br>Cytochrome P450, E-class, group I<br>Cytochrome P450, conserved site
LEMA_P08172 0.1	1.93	2.90	3.55E-02	8.11E+00	similar to aflatoxin B1 aldehyde reductase member 2	IPR023210	NADP-dependent oxidoreductase domain
LEMA_P01306 0.1	0.81	2.13	5.36E-03	3.93E+00	hypothetical protein	none	
LEMA_P02669 0.1	0.71	2.73	4.47E-02	2.60E+00	similar to FAD binding domain protein	none	

Supplementary Table S4. Genes of *Brassica napus* that are more highly expressed in *Lm*-inoculated cotyledons of 74-44 BL than those of Westar (score ≤ -2 × 10^4).

Gene Name	Expression	log2 Fold Change	Adjusted *p*-value	Score	InterPro ID	Description
BnaA01g17570D	2997.52	-3.41	2.35E-42	-4.26E+05	IPR000169	Cysteine peptidase, cysteine active site
					IPR000668	Peptidase C1A, papain C-terminal
					IPR013128	Peptidase C1A, papain
					IPR013201	Proteinase inhibitor I29, cathepsin propeptide
					IPR025661	Cysteine peptidase, asparagine active site
BnaA01g04000D	5722.37	-3.01	3.54E-11	-1.80E+05	IPR001096	Peptidase C13, legumain
BnaA09g52180D	7220.10	-2.14	2.34E-08	-1.18E+05	IPR000668	Peptidase C1A, papain C-terminal
					IPR012599	Peptidase C1A, propeptide
					IPR013128	Peptidase C1A, papain
					IPR015643	Peptidase C1A, cathepsin B
					IPR025660	Cysteine peptidase, histidine active site
BnaA07g20510D	2050.02	-4.29	9.36E-12	-9.70E+04	IPR006814	Photosystem II PsbR
BnaC04g46560D	18593.76	-2.05	5.95E-03	-8.50E+04	IPR003959	ATPase, AAA-type, core
BnaC04g10780D	13062.01	-2.30	6.65E-03	-6.53E+04	IPR001344	Chlorophyll A-B binding protein, plant
					IPR022796	Chlorophyll A-B binding protein
					IPR023329	Chlorophyll a/b binding protein domain
BnaC02g00130D	2502.42	-2.67	3.01E-09	-5.69E+04	IPR001461	Peptidase A1
					IPR001969	Peptidase aspartic, active site
					IPR021109	Aspartic peptidase
BnaA04g04110D	521.69	-2.56	3.25E-32	-4.21E+04	IPR004199	Beta galactosidase small chain/ domain 5
					IPR006101	Glycoside hydrolase, family 2
					IPR006102	Glycoside hydrolase, family 2, immunoglobulin-like beta-sandwich
					IPR006103	Glycoside hydrolase, family 2, TIM barrel
					IPR006104	Glycoside hydrolase, family 2, N-terminal
					IPR008979	Galactose-binding domain-like
					IPR011013	Galactose mutarotase-like domain
					IPR013781	Glycoside hydrolase, catalytic domain

					IPR013812	Glycoside hydrolase, family 2/20, immunoglobulin-like beta-sandwich domain
					IPR014718	Glycoside hydrolase-type carbohydrate-binding, subgroup
					IPR017853	Glycoside hydrolase, superfamily
					IPR023230	Glycoside hydrolase, family 2, conserved site
					IPR023232	Glycoside hydrolase, family 2, active site
BnaA09g13710D	5954.02	-2.48	1.41E-03	-4.21E+04	IPR001344	Chlorophyll A-B binding protein, plant
					IPR022796	Chlorophyll A-B binding protein
					IPR023329	Chlorophyll a/b binding protein domain
BnaA05g29390D	8516.26	-2.06	4.03E-03	-4.21E+04	IPR001344	Chlorophyll A-B binding protein, plant
					IPR022796	Chlorophyll A-B binding protein
					IPR023329	Chlorophyll a/b binding protein domain
BnaA10g25820D	518.97	-2.95	1.64E-27	-4.09E+04	IPR004193	Glycoside hydrolase, family 13, N-terminal
					IPR006047	Glycosyl hydrolase, family 13, catalytic domain
					IPR011839	Alpha-1,6-glucosidases, pullulanase-type
					IPR013781	Glycoside hydrolase, catalytic domain
					IPR013783	Immunoglobulin-like fold
					IPR014756	Immunoglobulin E-set
					IPR015902	Glycoside hydrolase, family 13
					IPR017853	Glycoside hydrolase, superfamily
					IPR024561	Alpha-1,6-glucosidases, pullulanase-type, C-terminal
BnaA02g00670D	2921.18	-2.37	1.89E-06	-3.96E+04	IPR001461	Peptidase A1
					IPR001969	Peptidase aspartic, active site
					IPR021109	Aspartic peptidase
BnaA03g44930D	603.62	-2.32	1.79E-28	-3.89E+04	IPR002579	Peptide methionine sulphoxide reductase MrsB
					IPR011057	Mss4-like
BnaA10g26060D	4072.11	-2.42	2.31E-04	-3.58E+04	IPR000583	Class II glutamine amidotransferase domain
					IPR002489	Glutamate synthase, alpha subunit, C-terminal
					IPR002932	Glutamate synthase, central-C
					IPR006982	Glutamate synthase, central-N
					IPR013785	Aldolase-type TIM barrel
					IPR017932	Glutamine amidotransferase type 2 domain

BnaA03g11710D	2828.36	-2.03	8.30E-07	-3.49E+04	IPR002922	Thiazole biosynthetic enzyme Thi4 family
BnaAnng14750D	2232.72	-2.28	2.74E-07	-3.34E+04	IPR001433	Oxidoreductase FAD/NAD(P)-binding
					IPR001709	Flavoprotein pyridine nucleotide cytochrome reductase
					IPR008333	Oxidoreductase, FAD-binding domain
					IPR012146	Ferredoxin-NADP+ reductase
					IPR015701	Ferredoxin Reductase
					IPR017927	Ferredoxin reductase-type FAD-binding domain
					IPR017938	Riboflavin synthase-like beta-barrel
BnaA09g01080D	5335.01	-2.04	1.26E-03	-3.16E+04	IPR003685	Photosystem I PsaD
BnaAnng00160D	3182.66	-2.73	3.39E-04	-3.02E+04	IPR001344	Chlorophyll A-B binding protein, plant
					IPR022796	Chlorophyll A-B binding protein
					IPR023329	Chlorophyll a/b binding protein domain
BnaC04g30870D	4634.79	-2.49	2.70E-03	-2.97E+04	IPR000894	Ribulose bisphosphate carboxylase small chain, domain
					IPR024680	Ribulose-1,5-bisphosphate carboxylase small subunit, N-terminal
					IPR024681	Ribulose bisphosphate carboxylase, small chain
BnaC08g49610D	1395.47	-2.92	5.17E-08	-2.97E+04	IPR002192	Pyruvate phosphate dikinase, PEP/pyruvate-binding
					IPR013815	ATP-grasp fold, subdomain 1
					IPR013816	ATP-grasp fold, subdomain 2
BnaA09g19870D	4432.54	-2.24	1.04E-03	-2.96E+04	IPR008797	Photosystem II PsbQ, oxygen evolving complex
					IPR023222	PsbQ-like domain
BnaAnng10080D	2261.06	-2.06	9.55E-07	-2.80E+04	IPR006652	Kelch repeat type 1
					IPR015915	Kelch-type beta propeller
					IPR015916	Galactose oxidase, beta-propeller
BnaA09g39860D	1271.72	-2.61	1.47E-08	-2.60E+04	IPR003823	Domain of unknown function CP12
BnaA07g12480D	1364.05	-3.41	2.88E-06	-2.58E+04	IPR002628	Photosystem II PsbO, manganese-stabilising
BnaA10g04360D	3681.08	-2.21	1.02E-03	-2.43E+04	IPR002683	Photosystem II PsbP, oxygen evolving complex
					IPR016123	Mog1/PsbP, alpha/beta/alpha sandwich
BnaC03g59520D	3386.56	-2.66	3.90E-03	-2.17E+04	IPR001344	Chlorophyll A-B binding protein, plant
					IPR022796	Chlorophyll A-B binding protein
					IPR023329	Chlorophyll a/b binding protein domain
BnaCnng24140D	4609.48	-2.20	7.27E-03	-2.17E+04	IPR001344	Chlorophyll A-B binding protein, plant

					IPR022796	Chlorophyll A-B binding protein
					IPR023329	Chlorophyll a/b binding protein domain
BnaA05g30550D	259.64	-2.66	1.03E-31	-2.14E+04	IPR001650	Helicase, C-terminal
					IPR011545	DNA/RNA helicase, DEAD/DEAH box type, N-terminal
					IPR014001	Helicase, superfamily 1/2, ATP-binding domain
					IPR014014	RNA helicase, DEAD-box type, Q motif
BnaA08g17660D	1196.77	-3.79	3.06E-05	-2.05E+04	IPR001344	Chlorophyll A-B binding protein, plant
					IPR022796	Chlorophyll A-B binding protein
					IPR023329	Chlorophyll a/b binding protein domain
BnaC05g04590D	2757.91	-2.21	4.83E-04	-2.02E+04	IPR002683	Photosystem II PsbP, oxygen evolving complex
					IPR016123	Mog1/PsbP, alpha/beta/alpha sandwich
BnaC07g11970D	354.31	-2.29	1.82E-25	-2.01E+04	IPR010920	Like-Sm (LSM) domain
					IPR019050	FDF domain
					IPR025609	Lsm14 N-terminal
					IPR025761	FFD box
					IPR025762	DFDF domain
					IPR025768	TFG box
BnaA09g52250D	1191.38	-2.65	4.40E-07	-2.01E+04	IPR000719	Protein kinase, catalytic domain
					IPR001245	Serine-threonine/tyrosine-protein kinase catalytic domain
					IPR002290	Serine/threonine- / dual specificity protein kinase, catalytic domain
					IPR008271	Serine/threonine-protein kinase, active site
					IPR009856	Light regulated Lir1
					IPR011009	Protein kinase-like domain
					IPR013320	Concanavalin A-like lectin/glucanase, subgroup
					IPR017441	Protein kinase, ATP binding site
					IPR020635	Tyrosine-protein kinase, catalytic domain
BnaC08g28700D	920.92	-2.83	2.03E-08	-2.00E+04	IPR001478	PDZ domain
					IPR004447	C-terminal-processing peptidase S41A
					IPR005151	Interphotoreceptor retinol-binding

Supplementary Table S5. Gene Ontology (GO) term enrichment of differentially expressed genes (DEGs) in *Brassica napus* that are more highly expressed in *Lm*-inoculated 74-44 BL than in inoculated Westar.

GO ID	GO Name	GO Category	FDR	*p*-Value	% Test	% Reference
GO:0006364	rRNA processing	BIOL. PROC.	2.02E-68	4.68E-71	11.45%	1.01%
GO:0019344	cysteine biosynthetic process	BIOL. PROC.	8.58E-57	3.68E-59	9.58%	0.85%
GO:0009773	photosynthetic electron transport in photosystem I	BIOL. PROC.	1.79E-54	9.05E-57	5.51%	0.15%
GO:0009637	response to blue light	BIOL. PROC.	5.32E-42	3.63E-44	6.39%	0.46%
GO:0010218	response to far red light	BIOL. PROC.	4.06E-41	2.91E-43	5.73%	0.35%
GO:0019288	isopentenyl diphosphate biosynthetic process, methylerythritol 4-phosphate pathway	BIOL. PROC.	1.53E-40	1.12E-42	7.60%	0.80%
GO:0010114	response to red light	BIOL. PROC.	3.90E-40	2.97E-42	5.84%	0.39%
GO:0009543	chloroplast thylakoid lumen	CELL. COMP.	7.56E-37	6.75E-39	4.30%	0.17%
GO:0035304	regulation of protein dephosphorylation	BIOL. PROC.	9.86E-37	8.90E-39	6.28%	0.56%
GO:0016168	chlorophyll binding	MOL. FUNCT.	1.12E-36	1.02E-38	3.74%	0.10%
GO:0010287	plastoglobule	CELL. COMP.	7.15E-30	8.03E-32	3.85%	0.20%
GO:0019252	starch biosynthetic process	BIOL. PROC.	1.08E-28	1.29E-30	5.73%	0.66%
GO:0018298	protein-chromophore linkage	BIOL. PROC.	9.34E-28	1.20E-29	3.08%	0.11%

GO:0010155	regulation of proton transport	BIOL. PROC.	1.88E-27	2.44E-29	4.07%	0.28%
GO:0000023	maltose metabolic process	BIOL. PROC.	3.97E-25	5.51E-27	4.63%	0.47%
GO:0010103	stomatal complex morphogenesis	BIOL. PROC.	1.36E-24	1.95E-26	4.74%	0.51%
GO:0048046	apoplast	CELL. COMP.	1.97E-24	2.87E-26	8.15%	1.77%
GO:0010027	thylakoid membrane organization	BIOL. PROC.	3.57E-22	5.51E-24	4.85%	0.63%
GO:0009654	photosystem II oxygen evolving complex	CELL. COMP.	6.75E-21	1.10E-22	2.09%	0.05%
GO:0015995	chlorophyll biosynthetic process	BIOL. PROC.	1.82E-20	3.02E-22	4.19%	0.50%
GO:0008266	poly(U) RNA binding	MOL. FUNCT.	8.93E-18	1.66E-19	1.98%	0.07%
GO:0009902	chloroplast relocation	BIOL. PROC.	3.27E-14	7.42E-16	2.86%	0.33%
GO:0070838	divalent metal ion transport	BIOL. PROC.	1.79E-13	4.26E-15	4.85%	1.13%
GO:0009735	response to cytokinin	BIOL. PROC.	2.64E-13	6.41E-15	4.74%	1.09%
GO:0009744	response to sucrose	BIOL. PROC.	8.31E-13	2.07E-14	4.07%	0.84%
GO:0043085	positive regulation of catalytic activity	BIOL. PROC.	4.28E-12	1.14E-13	3.85%	0.80%
GO:0009644	response to high light intensity	BIOL. PROC.	1.23E-11	3.34E-13	3.85%	0.83%
GO:0016556	mRNA modification	BIOL. PROC.	1.98E-11	5.45E-13	2.42%	0.31%
GO:0009538	photosystem I reaction center	CELL. COMP.	2.43E-11	6.73E-13	1.10%	0.03%

GO:0019761	glucosinolate biosynthetic process	BIOL. PROC.	5.61E-11	1.60E-12	3.41%	0.69%
GO:0009768	photosynthesis, light harvesting in photosystem I	BIOL. PROC.	5.82E-11	1.67E-12	0.88%	0.01%
GO:0009965	leaf morphogenesis	BIOL. PROC.	8.63E-11	2.50E-12	3.74%	0.84%
GO:0010310	regulation of hydrogen peroxide metabolic process	BIOL. PROC.	8.68E-11	2.52E-12	3.41%	0.70%
GO:0009595	detection of biotic stimulus	BIOL. PROC.	1.07E-10	3.13E-12	2.75%	0.45%
GO:0009409	response to cold	BIOL. PROC.	1.20E-10	3.54E-12	6.72%	2.42%
GO:0042742	defense response to bacterium	BIOL. PROC.	4.60E-10	1.38E-11	5.62%	1.86%
GO:0045893	positive regulation of transcription, DNA-templated	BIOL. PROC.	7.56E-10	2.31E-11	5.29%	1.71%
GO:0030093	chloroplast photosystem I	CELL. COMP.	1.35E-09	4.25E-11	0.66%	0.00%
GO:0010598	NAD(P)H dehydrogenase complex (plastoquinone)	CELL. COMP.	1.92E-09	6.12E-11	0.99%	0.03%
GO:0000165	MAPK cascade	BIOL. PROC.	3.02E-09	9.72E-11	3.85%	1.03%
GO:0016117	carotenoid biosynthetic process	BIOL. PROC.	4.30E-09	1.40E-10	2.53%	0.46%
GO:0009862	systemic acquired resistance, salicylic acid mediated signaling pathway	BIOL. PROC.	4.89E-09	1.60E-10	3.74%	0.99%
GO:0006636	unsaturated fatty acid biosynthetic process	BIOL. PROC.	7.19E-09	2.38E-10	2.09%	0.31%
GO:0042793	plastid transcription	BIOL. PROC.	1.36E-08	4.59E-10	1.76%	0.21%
GO:0019843	rRNA binding	MOL. FUNCT.	2.36E-08	8.12E-10	2.09%	0.33%

GO:0031348	negative regulation of defense response	BIOL. PROC.	3.18E-08	1.10E-09	3.96%	1.19%
GO:0004565	beta-galactosidase activity	MOL. FUNCT.	3.28E-08	1.14E-09	1.21%	0.08%
GO:0009697	salicylic acid biosynthetic process	BIOL. PROC.	6.73E-08	2.41E-09	3.19%	0.83%
GO:0010319	stromule	CELL. COMP.	7.34E-08	2.65E-09	1.54%	0.18%
GO:0003959	NADPH dehydrogenase activity	MOL. FUNCT.	1.63E-07	6.08E-09	0.77%	0.02%
GO:0009867	jasmonic acid mediated signaling pathway	BIOL. PROC.	3.78E-07	1.45E-08	3.74%	1.20%
GO:0019898	extrinsic component of membrane	CELL. COMP.	6.14E-07	2.41E-08	2.09%	0.41%
GO:0009780	photosynthetic NADP+ reduction	BIOL. PROC.	2.39E-06	9.58E-08	0.44%	0.00%
GO:0006655	phosphatidylglycerol biosynthetic process	BIOL. PROC.	3.26E-06	1.31E-07	1.54%	0.24%
GO:0010196	nonphotochemical quenching	BIOL. PROC.	4.07E-06	1.65E-07	0.66%	0.02%
GO:0031969	chloroplast membrane	CELL. COMP.	4.09E-06	1.66E-07	2.64%	0.73%
GO:0016226	iron-sulfur cluster assembly	BIOL. PROC.	9.42E-06	3.95E-07	1.76%	0.36%
GO:0010363	regulation of plant-type hypersensitive response	BIOL. PROC.	1.77E-05	7.54E-07	3.96%	1.56%
GO:0006612	protein targeting to membrane	BIOL. PROC.	2.48E-05	1.07E-06	4.19%	1.71%
GO:0042550	photosystem I stabilization	BIOL. PROC.	3.00E-05	1.30E-06	0.44%	0.01%

GO:0047100	glyceraldehyde-3-phosphate dehydrogenase (NADP+) (phosphorylating) activity	MOL. FUNCT.	3.00E-05	1.30E-06	0.44%	0.01%
GO:0042549	photosystem II stabilization	BIOL. PROC.	3.09E-05	1.34E-06	0.55%	0.02%
GO:0005762	mitochondrial large ribosomal subunit	CELL. COMP.	3.21E-05	1.41E-06	0.88%	0.08%
GO:0042335	cuticle development	BIOL. PROC.	5.20E-05	2.31E-06	1.21%	0.19%
GO:0032544	plastid translation	BIOL. PROC.	5.35E-05	2.38E-06	0.66%	0.04%
GO:0018316	peptide cross-linking via L-cystine	BIOL. PROC.	6.38E-05	2.87E-06	0.33%	0.00%
GO:0009783	photosystem II antenna complex	CELL. COMP.	6.67E-05	3.03E-06	0.44%	0.01%
GO:0030246	carbohydrate binding	MOL. FUNCT.	7.67E-05	3.51E-06	3.08%	1.13%
GO:0003735	structural constituent of ribosome	MOL. FUNCT.	8.24E-05	3.80E-06	3.85%	1.60%
GO:0009106	lipoate metabolic process	BIOL. PROC.	1.11E-04	5.19E-06	1.10%	0.17%
GO:0016984	ribulose-bisphosphate carboxylase activity	MOL. FUNCT.	1.22E-04	5.76E-06	0.55%	0.02%
GO:0000038	very long-chain fatty acid metabolic process	BIOL. PROC.	1.37E-04	6.58E-06	1.21%	0.21%
GO:0006546	glycine catabolic process	BIOL. PROC.	1.42E-04	6.86E-06	1.21%	0.21%
GO:0009782	photosystem I antenna complex	CELL. COMP.	1.48E-04	7.14E-06	0.33%	0.00%
GO:0009108	coenzyme biosynthetic process	BIOL. PROC.	1.59E-04	7.69E-06	2.31%	0.74%

GO:0071454	cellular response to anoxia	BIOL. PROC.	2.85E-04	1.42E-05	0.33%	0.00%
GO:0050661	NADP binding	MOL. FUNCT.	3.70E-04	1.85E-05	1.21%	0.24%
GO:0009505	plant-type cell wall	CELL. COMP.	5.17E-04	2.62E-05	2.75%	1.06%
GO:0006005	L-fucose biosynthetic process	BIOL. PROC.	7.55E-04	3.92E-05	0.33%	0.00%
GO:0050832	defense response to fungus	BIOL. PROC.	7.64E-04	3.97E-05	3.85%	1.78%
GO:0042744	hydrogen peroxide catabolic process	BIOL. PROC.	8.28E-04	4.34E-05	1.87%	0.58%
GO:0005528	FK506 binding	MOL. FUNCT.	9.23E-04	4.89E-05	0.66%	0.07%
GO:0005247	voltage-gated chloride channel activity	MOL. FUNCT.	9.41E-04	5.01E-05	0.44%	0.02%
GO:0009533	chloroplast stromal thylakoid	CELL. COMP.	1.11E-03	5.97E-05	0.44%	0.02%
GO:0090042	tubulin deacetylation	BIOL. PROC.	1.45E-03	8.07E-05	0.22%	0.00%
GO:0051721	protein phosphatase 2A binding	MOL. FUNCT.	1.45E-03	8.07E-05	0.22%	0.00%
GO:0042903	tubulin deacetylase activity	MOL. FUNCT.	1.45E-03	8.07E-05	0.22%	0.00%
GO:0043014	alpha-tubulin binding	MOL. FUNCT.	1.45E-03	8.07E-05	0.22%	0.00%
GO:0034707	chloride channel complex	CELL. COMP.	1.49E-03	8.27E-05	0.44%	0.02%
GO:0010242	oxygen evolving activity	MOL. FUNCT.	1.49E-03	8.28E-05	0.33%	0.01%
GO:0043086	negative regulation of catalytic activity	BIOL. PROC.	1.58E-03	8.82E-05	2.31%	0.88%

GO:0031012	extracellular matrix	CELL. COMP.	1.73E-03	9.75E-05	0.77%	0.11%
GO:0006833	water transport	BIOL. PROC.	1.80E-03	1.03E-04	1.98%	0.69%
GO:0000413	protein peptidyl-prolyl isomerization	BIOL. PROC.	1.91E-03	1.09E-04	0.99%	0.19%
GO:0003755	peptidyl-prolyl cis-trans isomerase activity	MOL. FUNCT.	1.91E-03	1.09E-04	0.99%	0.19%
GO:0009508	plastid chromosome	CELL. COMP.	2.02E-03	1.16E-04	0.55%	0.05%
GO:1902476	chloride transmembrane transport	BIOL. PROC.	2.22E-03	1.29E-04	0.44%	0.02%
GO:0080153	negative regulation of reductive pentose-phosphate cycle	BIOL. PROC.	2.55E-03	1.50E-04	0.33%	0.01%
GO:0050577	GDP-L-fucose synthase activity	MOL. FUNCT.	2.55E-03	1.50E-04	0.33%	0.01%
GO:0015996	chlorophyll catabolic process	BIOL. PROC.	2.57E-03	1.51E-04	0.99%	0.20%
GO:0009737	response to abscisic acid	BIOL. PROC.	2.75E-03	1.62E-04	5.07%	2.79%
GO:0050821	protein stabilization	BIOL. PROC.	2.84E-03	1.68E-04	0.44%	0.03%
GO:0045036	protein targeting to chloroplast	BIOL. PROC.	3.14E-03	1.88E-04	1.10%	0.26%
GO:0004040	amidase activity	MOL. FUNCT.	3.22E-03	1.93E-04	0.33%	0.01%
GO:0045454	cell redox homeostasis	BIOL. PROC.	3.37E-03	2.03E-04	1.65%	0.54%
GO:0042631	cellular response to water deprivation	BIOL. PROC.	3.49E-03	2.12E-04	1.21%	0.31%
GO:0005509	calcium ion binding	MOL. FUNCT.	3.49E-03	2.11E-04	2.53%	1.05%

GO:0043481	anthocyanin accumulation in tissues in response to UV light	BIOL. PROC.	3.83E-03	2.35E-04	1.54%	0.49%
GO:0010189	vitamin E biosynthetic process	BIOL. PROC.	3.89E-03	2.42E-04	0.44%	0.03%
GO:0043864	indoleacetamide hydrolase activity	MOL. FUNCT.	3.89E-03	2.41E-04	0.22%	0.00%
GO:0031679	NADH dehydrogenase (plastoquinone) activity	MOL. FUNCT.	3.89E-03	2.41E-04	0.22%	0.00%
GO:0004857	enzyme inhibitor activity	MOL. FUNCT.	4.22E-03	2.66E-04	1.98%	0.75%
GO:0015250	water channel activity	MOL. FUNCT.	4.65E-03	2.96E-04	0.77%	0.13%
GO:0010019	chloroplast-nucleus signaling pathway	BIOL. PROC.	4.74E-03	3.04E-04	0.44%	0.03%
GO:0080170	hydrogen peroxide transmembrane transport	BIOL. PROC.	4.74E-03	3.04E-04	0.33%	0.01%
GO:0016151	nickel cation binding	MOL. FUNCT.	4.74E-03	3.04E-04	0.33%	0.01%
GO:0016709	oxidoreductase activity, acting on paired donors, with incorporation or reduction of molecular oxygen, NAD(P)H as one donor, and incorporation of one atom of oxygen	MOL. FUNCT.	5.04E-03	3.23E-04	1.10%	0.28%
GO:0045735	nutrient reservoir activity	MOL. FUNCT.	5.72E-03	3.69E-04	0.88%	0.18%
GO:0000311	plastid large ribosomal subunit	CELL. COMP.	5.79E-03	3.75E-04	0.44%	0.03%
GO:0030091	protein repair	BIOL. PROC.	5.83E-03	3.79E-04	0.55%	0.06%
GO:0009269	response to desiccation	BIOL. PROC.	6.09E-03	3.97E-04	0.77%	0.14%

GO:0097339	glycolate transmembrane transport	BIOL. PROC.	7.20E-03	4.78E-04	0.22%	0.00%
GO:1901975	glycerate transmembrane transport	BIOL. PROC.	7.20E-03	4.78E-04	0.22%	0.00%
GO:0043879	glycolate transmembrane transporter activity	MOL. FUNCT.	7.20E-03	4.78E-04	0.22%	0.00%
GO:0008531	riboflavin kinase activity	MOL. FUNCT.	7.20E-03	4.78E-04	0.22%	0.00%
GO:0009671	nitrate:proton symporter activity	MOL. FUNCT.	7.20E-03	4.78E-04	0.22%	0.00%
GO:0010301	xanthoxin dehydrogenase activity	MOL. FUNCT.	7.20E-03	4.78E-04	0.22%	0.00%
GO:0045550	geranylgeranyl reductase activity	MOL. FUNCT.	7.20E-03	4.78E-04	0.22%	0.00%
GO:1901974	glycerate transmembrane transporter activity	MOL. FUNCT.	7.20E-03	4.78E-04	0.22%	0.00%
GO:0006354	DNA-templated transcription, elongation	BIOL. PROC.	7.33E-03	4.88E-04	1.21%	0.35%
GO:0009772	photosynthetic electron transport in photosystem II	BIOL. PROC.	7.55E-03	5.04E-04	0.44%	0.04%
GO:0003958	NADPH-hemoprotein reductase activity	MOL. FUNCT.	7.55E-03	5.04E-04	0.44%	0.04%
GO:0010118	stomatal movement	BIOL. PROC.	9.18E-03	6.17E-04	1.43%	0.48%
GO:0015035	protein disulfide oxidoreductase activity	MOL. FUNCT.	9.39E-03	6.33E-04	1.10%	0.30%
GO:0009695	jasmonic acid biosynthetic process	BIOL. PROC.	1.03E-02	7.00E-04	1.54%	0.55%
GO:0016671	oxidoreductase activity, acting on a sulfur group of donors, disulfide as acceptor	MOL. FUNCT.	1.12E-02	7.61E-04	0.66%	0.11%

GO:0071277	cellular response to calcium ion	BIOL. PROC.	1.15E-02	7.92E-04	0.22%	0.00%
GO:0050278	sedoheptulose-bisphosphatase activity	MOL. FUNCT.	1.15E-02	7.92E-04	0.22%	0.00%
GO:0008974	phosphoribulokinase activity	MOL. FUNCT.	1.15E-02	7.92E-04	0.22%	0.00%
GO:0008465	glycerate dehydrogenase activity	MOL. FUNCT.	1.15E-02	7.92E-04	0.22%	0.00%
GO:0046406	magnesium protoporphyrin IX methyltransferase activity	MOL. FUNCT.	1.15E-02	7.92E-04	0.22%	0.00%
GO:0052689	carboxylic ester hydrolase activity	MOL. FUNCT.	1.22E-02	8.44E-04	2.53%	1.16%
GO:0055070	copper ion homeostasis	BIOL. PROC.	1.23E-02	8.50E-04	0.44%	0.04%
GO:0042351	'de novo' GDP-L-fucose biosynthetic process	BIOL. PROC.	1.23E-02	8.53E-04	0.33%	0.02%
GO:0009051	pentose-phosphate shunt, oxidative branch	BIOL. PROC.	1.23E-02	8.53E-04	0.33%	0.02%
GO:0051537	2 iron, 2 sulfur cluster binding	MOL. FUNCT.	1.23E-02	8.53E-04	0.88%	0.21%
GO:0031408	oxylipin biosynthetic process	BIOL. PROC.	1.25E-02	8.76E-04	0.77%	0.16%
GO:0016628	oxidoreductase activity, acting on the CH-CH group of donors, NAD or NADP as acceptor	MOL. FUNCT.	1.38E-02	9.72E-04	0.77%	0.16%
GO:0004345	glucose-6-phosphate dehydrogenase activity	MOL. FUNCT.	1.39E-02	9.81E-04	0.33%	0.02%
GO:0050162	oxalate oxidase activity	MOL. FUNCT.	1.39E-02	9.81E-04	0.33%	0.02%
GO:0009740	gibberellic acid mediated signaling pathway	BIOL. PROC.	1.55E-02	1.10E-03	1.21%	0.39%

GO:0006048	UDP-N-acetylglucosamine biosynthetic process	BIOL. PROC.	1.56E-02	1.12E-03	0.33%	0.02%
GO:0008106	alcohol dehydrogenase (NADP+) activity	MOL. FUNCT.	1.56E-02	1.12E-03	0.33%	0.02%
GO:0045490	pectin catabolic process	BIOL. PROC.	1.57E-02	1.13E-03	1.10%	0.33%
GO:0019747	regulation of isoprenoid metabolic process	BIOL. PROC.	1.60E-02	1.16E-03	0.44%	0.05%
GO:0017148	negative regulation of translation	BIOL. PROC.	1.60E-02	1.16E-03	0.44%	0.05%
GO:0009344	nitrite reductase complex [NAD(P)H]	CELL. COMP.	1.62E-02	1.18E-03	0.22%	0.00%
GO:0031409	pigment binding	MOL. FUNCT.	1.62E-02	1.18E-03	0.22%	0.00%
GO:0009496	plastoquinol--plastocyanin reductase activity	MOL. FUNCT.	1.62E-02	1.18E-03	0.22%	0.00%
GO:0045156	electron transporter, transferring electrons within the cyclic electron transport pathway of photosynthesis activity	MOL. FUNCT.	1.74E-02	1.27E-03	0.33%	0.02%
GO:0010304	PSII associated light-harvesting complex II catabolic process	BIOL. PROC.	1.94E-02	1.43E-03	0.55%	0.09%
GO:0042132	fructose 1,6-bisphosphate 1-phosphatase activity	MOL. FUNCT.	2.18E-02	1.61E-03	0.33%	0.02%
GO:1902066	regulation of cell wall pectin metabolic process	BIOL. PROC.	2.21E-02	1.64E-03	0.22%	0.00%
GO:0009769	photosynthesis, light harvesting in photosystem II	BIOL. PROC.	2.21E-02	1.64E-03	0.22%	0.00%
GO:0048487	beta-tubulin binding	MOL. FUNCT.	2.21E-02	1.64E-03	0.22%	0.00%
GO:0008124	4-alpha-hydroxytetrahydrobiopterin dehydratase activity	MOL. FUNCT.	2.21E-02	1.64E-03	0.22%	0.00%

GO:0009073	aromatic amino acid family biosynthetic process	BIOL. PROC.	2.37E-02	1.78E-03	1.21%	0.41%
GO:0046688	response to copper ion	BIOL. PROC.	2.49E-02	1.87E-03	0.44%	0.05%
GO:0051287	NAD binding	MOL. FUNCT.	2.57E-02	1.93E-03	0.88%	0.24%
GO:0016122	xanthophyll metabolic process	BIOL. PROC.	2.63E-02	1.99E-03	0.44%	0.05%
GO:0004332	fructose-bisphosphate aldolase activity	MOL. FUNCT.	2.65E-02	2.00E-03	0.33%	0.02%
GO:0009279	cell outer membrane	CELL. COMP.	2.85E-02	2.18E-03	0.22%	0.01%
GO:0045252	oxoglutarate dehydrogenase complex	CELL. COMP.	2.85E-02	2.18E-03	0.22%	0.01%
GO:0004149	dihydrolipoyllysine-residue succinyltransferase activity	MOL. FUNCT.	2.85E-02	2.18E-03	0.22%	0.01%
GO:0051920	peroxiredoxin activity	MOL. FUNCT.	2.89E-02	2.22E-03	0.33%	0.03%
GO:0030145	manganese ion binding	MOL. FUNCT.	2.92E-02	2.24E-03	0.66%	0.14%
GO:0016575	histone deacetylation	BIOL. PROC.	2.93E-02	2.25E-03	0.44%	0.06%
GO:0009750	response to fructose	BIOL. PROC.	3.14E-02	2.44E-03	1.54%	0.63%
GO:0048359	mucilage metabolic process involved in seed coat development	BIOL. PROC.	3.26E-02	2.54E-03	0.44%	0.06%
GO:0006066	alcohol metabolic process	BIOL. PROC.	3.45E-02	2.70E-03	1.87%	0.84%
GO:0010270	photosystem II oxygen evolving complex assembly	BIOL. PROC.	3.54E-02	2.79E-03	0.22%	0.01%
GO:0004560	alpha-L-fucosidase activity	MOL. FUNCT.	3.54E-02	2.79E-03	0.22%	0.01%

GO:0080093	regulation of photorespiration	BIOL. PROC.	4.32E-02	3.46E-03	0.22%	0.01%
GO:0031998	regulation of fatty acid beta-oxidation	BIOL. PROC.	4.32E-02	3.46E-03	0.22%	0.01%
GO:0010617	circadian regulation of calcium ion oscillation	BIOL. PROC.	4.32E-02	3.46E-03	0.22%	0.01%
GO:0010258	NADH dehydrogenase complex (plastoquinone) assembly	BIOL. PROC.	4.32E-02	3.46E-03	0.22%	0.01%
GO:0009517	PSII associated light-harvesting complex II	CELL. COMP.	4.32E-02	3.46E-03	0.22%	0.01%
GO:0004615	phosphomannomutase activity	MOL. FUNCT.	4.32E-02	3.46E-03	0.22%	0.01%
GO:0006662	glycerol ether metabolic process	BIOL. PROC.	4.39E-02	3.53E-03	0.55%	0.11%

Supplementary Table S6. KEGG annotation of differentially expressed genes (DEGs) in *Brassica napus* that are more highly expressed in *Lm*-inoculated 74-44 BL than in inoculated Westar (scores > 1.0×10^3).

Gene ID	Expression	log2 Fold Change	adjusted p-value	Score	KEGG pathway
BnaA04g04110D	521.69	-2.56	3.25E-32	-4.21E+04	Galactose metabolism<br>Other glycan degradation<br>Glycosaminoglycan degradation<br>Sphingolipid metabolism<br>Glycosphingolipid biosynthesis - ganglio series
BnaA03g11710D	2828.36	-2.03	8.30E-07	-3.49E+04	Thiamine metabolism
BnaAnng14750D	2232.72	-2.28	2.74E-07	-3.34E+04	Photosynthesis

BnaC04g30870D	4634.79	-2.49	2.70E-03	-2.97E+04	Glyoxylate and dicarboxylate metabolism<br>Carbon fixation in photosynthetic organisms
BnaA06g17710D	1193.48	-2.33	1.79E-06	-1.60E+04	Starch and sucrose metabolism
BnaA07g28910D	677.05	-2.34	1.50E-10	-1.56E+04	Amino sugar and nucleotide sugar metabolism
BnaAnng36210D	3591.63	-2.71	3.59E-02	-1.40E+04	Glyoxylate and dicarboxylate metabolism<br>Carbon fixation in photosynthetic organisms
BnaA03g49440D	879.45	-2.44	4.55E-07	-1.36E+04	Taurine and hypotaurine metabolism<br>Cyanoamino acid metabolism<br>Glutathione metabolism
BnaC02g00740D	1288.39	-2.20	4.95E-05	-1.22E+04	Citrate cycle (TCA cycle)<br>Cysteine and methionine metabolism<br>Pyruvate metabolism<br>Glyoxylate and dicarboxylate metabolism<br>Carbon fixation in photosynthetic organisms
BnaA01g15640D	747.11	-3.22	1.63E-05	-1.15E+04	Glycolysis / Gluconeogenesis<br>Pentose phosphate pathway<br>Fructose and mannose metabolism<br>Carbon fixation in photosynthetic organisms
BnaA04g08570D	1324.68	-3.50	3.29E-03	-1.15E+04	Glyoxylate and dicarboxylate metabolism<br>Carbon fixation in photosynthetic organisms

BnaA05g34260D	713.53	-2.53	4.88E-07	-1.14E+04	Pyruvate metabolism<br>Carbon fixation in photosynthetic organisms
BnaAnng15070D	222.60	-2.47	2.18E-18	-9.72E+03	Aminoacyl-tRNA biosynthesis
BnaA09g46780D	1218.95	-2.55	8.32E-04	-9.57E+03	Glycolysis / Gluconeogenesis<br>Carbon fixation in photosynthetic organisms
BnaC01g22950D	894.64	-2.15	1.44E-05	-9.30E+03	Purine metabolism<br>Monobactam biosynthesis<br>Selenocompound metabolism<br>Sulfur metabolism
BnaC07g41840D	823.34	-2.19	1.13E-05	-8.92E+03	Taurine and hypotaurine metabolism<br>Cyanoamino acid metabolism<br>Glutathione metabolism
BnaC06g31970D	628.47	-2.57	3.50E-06	-8.82E+03	Amino sugar and nucleotide sugar metabolism
BnaA08g31180D	556.98	-2.62	1.06E-06	-8.70E+03	Photosynthesis
BnaA02g33920D	996.13	-2.25	1.90E-04	-8.36E+03	Galactose metabolism<br>Other glycan degradation<br>Glycosaminoglycan degradation<br>Sphingolipid metabolism<br>Glycosphingolipid biosynthesis - ganglio series
BnaA01g21620D	270.07	-2.59	3.29E-11	-7.34E+03	Nitrogen metabolism
BnaA09g16630D	334.12	-2.04	2.54E-11	-7.22E+03	Cysteine and methionine metabolism
BnaA08g16820D	1138.74	-2.31	2.30E-03	-6.94E+03	Glycolysis / Gluconeogenesis<br>Pentose phosphate pathway

					Fructose and mannose metabolism<br>Carbon fixation in photosynthetic organisms
BnaC05g39570D	1636.46	-2.28	1.45E-02	-6.87E+03	Galactose metabolism<br>Other glycan degradation<br>Glycosaminoglycan degradation<br>Sphingolipid metabolism<br>Glycosphingolipid biosynthesis - ganglio series
BnaA07g38250D	1503.31	-2.07	6.23E-03	-6.85E+03	Galactose metabolism<br>Other glycan degradation<br>Glycosaminoglycan degradation<br>Sphingolipid metabolism<br>Glycosphingolipid biosynthesis - ganglio series
BnaC05g09210D	1589.05	-2.05	1.11E-02	-6.38E+03	Glycolysis / Gluconeogenesis<br>Carbon fixation in photosynthetic organisms
BnaC06g17920D	570.18	-2.51	5.23E-05	-6.14E+03	Porphyrin and chlorophyll metabolism
BnaC02g42730D	826.28	-2.19	7.08E-04	-5.71E+03	Galactose metabolism<br>Other glycan degradation<br>Glycosaminoglycan degradation<br>Sphingolipid metabolism<br>Glycosphingolipid biosynthesis - ganglio series
BnaC07g14870D	718.05	-2.01	1.39E-04	-5.58E+03	Photosynthesis
BnaC08g40850D	803.37	-2.33	1.10E-03	-5.54E+03	Glycolysis / Gluconeogenesis<br>Carbon fixation in photosynthetic organisms

BnaA06g32740D	461.77	-2.67	3.82E-05	-5.45E+03	Glycolysis / Gluconeogenesis<br>Carbon fixation in photosynthetic organisms
BnaC08g19150D	598.40	-2.25	1.32E-04	-5.22E+03	Photosynthesis
BnaC08g23140D	158.41	-3.18	5.38E-11	-5.17E+03	Porphyrin and chlorophyll metabolism
BnaC05g24200D	656.87	-2.41	1.00E-03	-4.75E+03	Carbon fixation in photosynthetic organisms
BnaC03g30230D	434.80	-2.30	2.36E-05	-4.63E+03	Photosynthesis
BnaC08g15120D	94.97	-3.26	2.24E-15	-4.53E+03	Citrate cycle (TCA cycle)<br>Lysine degradation<br>Tryptophan metabolism
BnaC04g34390D	879.31	-2.97	1.85E-02	-4.53E+03	Glyoxylate and dicarboxylate metabolism<br>Carbon fixation in photosynthetic organisms
BnaC01g18640D	805.47	-2.13	2.57E-03	-4.44E+03	Pyrimidine metabolism
BnaC01g27750D	361.88	-2.28	4.56E-06	-4.40E+03	Nitrogen metabolism
BnaC05g39340D	489.13	-2.67	4.43E-04	-4.38E+03	Pentose and glucuronate interconversions
BnaA07g31590D	757.66	-2.41	5.06E-03	-4.19E+03	Terpenoid backbone biosynthesis
BnaA06g26720D	397.10	-2.40	6.85E-05	-3.96E+03	Steroid biosynthesis<br>Sesquiterpenoid and triterpenoid biosynthesis
BnaC08g26740D	527.49	-2.23	5.34E-04	-3.85E+03	Glycolysis / Gluconeogenesis<br>Pentose phosphate pathway<br>Fructose and mannose metabolism

					Carbon fixation in photosynthetic organisms
BnaA09g54020D	804.50	-2.18	8.04E-03	-3.67E+03	Carbon fixation in photosynthetic organisms
BnaA06g04940D	191.87	-2.17	3.53E-09	-3.52E+03	Carotenoid biosynthesis
BnaA03g31380D	81.73	-3.07	2.80E-13	-3.15E+03	Amino sugar and nucleotide sugar metabolism
BnaA03g13290D	233.46	-2.69	1.28E-05	-3.07E+03	Folate biosynthesis
BnaC03g16100D	263.13	-2.17	8.34E-06	-2.89E+03	Folate biosynthesis
BnaC03g29830D	100.77	-2.62	1.17E-11	-2.89E+03	Aminoacyl-tRNA biosynthesis
BnaC04g23180D	70.33	-2.62	1.39E-15	-2.74E+03	Glycolysis / Gluconeogenesis<br>Purine metabolism<br>Pyruvate metabolism
BnaA01g06700D	78.24	-2.40	3.30E-14	-2.53E+03	Glycerolipid metabolism<br>Glycerophospholipid metabolism<br>Phosphatidylinositol signaling system
BnaA01g37210D	521.56	-2.42	1.21E-02	-2.42E+03	Galactose metabolism<br>Other glycan degradation<br>Glycosaminoglycan degradation<br>Sphingolipid metabolism<br>Glycosphingolipid biosynthesis - ganglio series
BnaA08g25090D	73.22	-2.83	2.83E-12	-2.39E+03	Citrate cycle (TCA cycle)<br>Lysine degradation<br>Tryptophan metabolism
BnaC03g66720D	189.18	-2.52	1.50E-05	-2.30E+03	Pentose and glucuronate interconversions

BnaA06g19490D	289.52	-2.14	2.72E-04	-2.21E+03	Glutathione metabolism
BnaC04g38330D	237.40	-2.02	1.71E-04	-1.81E+03	Fatty acid biosynthesis<br>Fatty acid elongation
BnaC04g25260D	157.79	-2.56	4.74E-05	-1.75E+03	Glycolysis / Gluconeogenesis<br>Pentose phosphate pathway<br>Fructose and mannose metabolism<br>Carbon fixation in photosynthetic organisms
BnaC01g17000D	264.54	-2.02	6.08E-04	-1.72E+03	Porphyrin and chlorophyll metabolism
BnaC03g19540D	69.09	-2.91	3.97E-09	-1.69E+03	Fatty acid degradation<br>beta-Alanine metabolism<br>alpha-Linolenic acid metabolism<br>Propanoate metabolism
BnaA01g31480D	223.94	-2.64	2.89E-03	-1.50E+03	Pentose and glucuronate interconversions
BnaC06g35410D	372.19	-2.29	1.80E-02	-1.49E+03	Terpenoid backbone biosynthesis
BnaC02g29350D	138.81	-2.42	2.48E-04	-1.21E+03	Other glycan degradation<br>Sphingolipid metabolism
BnaA02g07560D	78.38	-2.10	1.04E-07	-1.15E+03	Inositol phosphate metabolism<br>Phosphatidylinositol signaling system
BnaAnng17200D	217.03	-2.06	2.88E-03	-1.14E+03	Glycolysis / Gluconeogenesis<br>Pentose phosphate pathway<br>Fructose and mannose metabolism<br>Carbon fixation in photosynthetic organisms
BnaC03g03330D	69.01	-2.29	8.27E-08	-1.12E+03	Glycerolipid metabolism<br>Glycerophospholipid metabolism<br>Phosphatidylinositol signaling system

BnaC01g37460D	255.98	-2.31	1.34E-02	-1.11E+03	Galactose metabolism<br>Other glycan degradation<br>Glycosaminoglycan degradation<br>Sphingolipid metabolism<br>Glycosphingolipid biosynthesis - ganglio series
BnaA03g17000D	48.72	-2.95	2.59E-08	-1.09E+03	Phenylpropanoid biosynthesis
BnaA03g22180D	88.24	-2.64	4.21E-05	-1.02E+03	Galactose metabolism<br>Other glycan degradation<br>Glycosaminoglycan degradation<br>Sphingolipid metabolism<br>Glycosphingolipid biosynthesis - ganglio series

Supplementary Table S7. Genes of *Brassica napus* that are more highly expressed in *Lm*-inoculated cotyledons of Westar than in those of 74-44 BL (scores > 2×10^4).

Gene Name	Expression	log2 Fold Change	Adjusted *p*-value	Score	InterPro ID	Description
BnaC09g20030D	3410.28	2.20	1.13E-28	2.09E+05	IPR006213<br>IPR006214	Bax inhibitor 1, conserved site<br>Bax inhibitor 1-related
BnaA04g06220D	1236.46	2.82	6.75E-33	1.12E+05	IPR006895<br>IPR006896<br>IPR006900<br>IPR007123<br>IPR012990	Zinc finger, Sec23/Sec24-type<br>Sec23/Sec24, trunk domain<br>Sec23/Sec24, helical domain<br>Gelsolin domain<br>Sec23/Sec24 beta-sandwich
BnaCnng58090D	2353.53	2.39	2.71E-16	8.75E+04	IPR013989	Development/cell death domain.
BnaC08g42820D	767.75	4.53	7.76E-26	8.73E+04	IPR013126<br>IPR018181	Heat shock protein 70 family<br>Heat shock protein 70, conserved site
BnaAnng22050D	1884.66	2.62	5.24E-17	8.04E+04	IPR004839	Aminotransferase, class I/classII

					IPR015421	Pyridoxal phosphate-dependent transferase, major region, subdomain 1
					IPR015422	Pyridoxal phosphate-dependent transferase, major region, subdomain 2
					IPR015424	Pyridoxal phosphate-dependent transferase
BnaA08g26550D	971.89	2.67	1.70E-31	8.00E+04	IPR005225	Small GTP-binding protein domain
					IPR006687	Small GTPase superfamily, SAR1-type
					IPR006689	Small GTPase superfamily, ARF/SAR type
					IPR024156	Small GTPase superfamily, ARF type
BnaA06g05280D	1986.35	2.35	2.64E-15	6.79E+04	IPR005225	Small GTP-binding protein domain
					IPR006687	Small GTPase superfamily, SAR1-type
					IPR006689	Small GTPase superfamily, ARF/SAR type
					IPR024156	Small GTPase superfamily, ARF type
BnaA06g31440D	866.61	3.55	1.64E-21	6.40E+04	IPR003593	AAA+ ATPase domain
					IPR003959	ATPase, AAA-type, core
					IPR025753	AAA-type ATPase, N-terminal domain
BnaC08g18340D	954.57	3.24	1.15E-20	6.16E+04	IPR001680	WD40 repeat
					IPR015943	WD40/YVTN repeat-like-containing domain
					IPR017986	WD40-repeat-containing domain
					IPR019775	WD40 repeat, conserved site
BnaC06g24690D	3099.53	2.28	1.99E-09	6.14E+04	IPR003579	Small GTPase superfamily, Rab type
					IPR005225	Small GTP-binding protein domain
					IPR006687	Small GTPase superfamily, SAR1-type
					IPR006689	Small GTPase superfamily, ARF/SAR type
					IPR024156	Small GTPase superfamily, ARF type
BnaA09g26960D	2590.93	2.07	6.08E-11	5.47E+04	IPR002208	SecY/SEC61-alpha family
					IPR019561	Translocon Sec61/SecY, plug domain
					IPR023201	SecY subunit domain
BnaC03g44640D	850.32	2.14	3.08E-30	5.37E+04	IPR001179	Peptidyl-prolyl cis-trans isomerase, FKBP-type, domain
					IPR011990	Tetratricopeptide-like helical
					IPR013026	Tetratricopeptide repeat-containing domain

					IPR019734	Tetratricopeptide repeat
					IPR023566	Peptidyl-prolyl cis-trans isomerase, FKBP-type
BnaA05g20940D	604.35	6.18	6.22E-15	5.31E+04	IPR004314	Domain of unknown function DUF239
					IPR025521	Domain of unknown function DUF4409
BnaA07g09950D	983.34	2.13	4.51E-26	5.30E+04	IPR003579	Small GTPase superfamily, Rab type
					IPR005225	Small GTP-binding protein domain
					IPR006687	Small GTPase superfamily, SAR1-type
					IPR006689	Small GTPase superfamily, ARF/SAR type
					IPR024156	Small GTPase superfamily, ARF type
BnaA05g16820D	220.73	5.24	3.74E-45	5.14E+04	IPR001757	Cation-transporting P-type ATPase
					IPR004014	Cation-transporting P-type ATPase, N-terminal
					IPR006068	Cation-transporting P-type ATPase, C-terminal
					IPR006408	Calcium-transporting P-type ATPase, subfamily IIB
					IPR008250	P-type ATPase, A domain
					IPR018303	P-type ATPase, phosphorylation site
					IPR023214	HAD-like domain
					IPR023298	P-type ATPase, transmembrane domain
					IPR023299	P-type ATPase, cytoplasmic domain N
BnaC03g41930D	847.71	2.16	6.09E-25	4.42E+04	IPR001382	Glycoside hydrolase, family 47
BnaCnng06680D	1100.64	2.28	1.06E-15	3.75E+04	IPR005225	Small GTP-binding protein domain
					IPR006687	Small GTPase superfamily, SAR1-type
					IPR006689	Small GTPase superfamily, ARF/SAR type
					IPR024156	Small GTPase superfamily, ARF type
BnaC03g39420D	417.07	2.37	2.23E-37	3.63E+04	IPR001680	WD40 repeat
					IPR006692	Coatomer, W
					IPR015943	WD40/YVTN repeat-like-containing domain
					IPR016453	Coatomer beta' subunit (COPB2)
					IPR017986	WD40-repeat-containing domain
					IPR020472	G-protein beta WD-40 repeat
BnaA07g34510D	449.54	3.01	5.05E-27	3.56E+04	IPR004131	Pyrophosphate-energised proton pump
BnaA09g05230D	746.34	2.25	3.04E-19	3.10E+04	IPR008429	Cleft lip and palate transmembrane 1

BnaA03g00370D	346.01	3.02	3.74E-30	3.07E+04	IPR007305	Vesicle transport protein, Got1/SFT2-like
BnaC03g07130D	999.98	2.79	1.08E-11	3.06E+04	IPR004901	Reversibly glycosylated polypeptide family
BnaA01g07540D	610.05	2.38	1.84E-18	2.58E+04	IPR007599	Derlin
BnaC08g25990D	545.89	2.91	8.40E-16	2.40E+04	IPR003439	ABC transporter-like
					IPR003593	AAA+ ATPase domain
					IPR013525	ABC-2 type transporter
					IPR017871	ABC transporter, conserved site
BnaA09g32460D	744.70	2.54	3.00E-13	2.37E+04	IPR011989	Armadillo-like helical
					IPR013918	Nucleotide exchange factor Fes1
					IPR016024	Armadillo-type fold
BnaC06g41450D	1789.63	2.16	4.69E-06	2.06E+04	IPR003439	ABC transporter-like
					IPR003593	AAA+ ATPase domain
					IPR017871	ABC transporter, conserved site

Supplementary Table S8. GO term enrichment of DEGs in *Brassica napus* that are more highly expressed in *Lm*- inoculated Westar than in inoculated 74-44 BL.

GO ID	GO Name	GO Category	FDR	*p*-Value	% Test	% Reference
GO:0042542	response to hydrogen peroxide	BIOL. PROC.	2.31E-08	1.11E-11	4.06%	0.75%
GO:0006457	protein folding	BIOL. PROC.	2.31E-08	1.27E-11	5.31%	1.29%
GO:0034976	response to endoplasmic reticulum stress	BIOL. PROC.	3.77E-08	2.91E-11	5.78%	1.56%
GO:0009644	response to high light intensity	BIOL. PROC.	4.19E-07	6.00E-10	3.91%	0.84%
GO:0006984	ER-nucleus signaling pathway	BIOL. PROC.	5.21E-04	3.16E-06	0.94%	0.06%
GO:0006888	endoplasmic reticulum to Golgi vesicle-mediated transport	BIOL. PROC.	9.53E-04	6.98E-06	2.19%	0.50%
GO:0006499	N-terminal protein myristoylation	BIOL. PROC.	9.53E-04	7.14E-06	2.19%	0.50%
GO:0034605	cellular response to heat	BIOL. PROC.	4.59E-03	4.29E-05	0.94%	0.09%

GO:0006094	gluconeogenesis	BIOL. PROC.	5.10E-03	4.94E-05	2.66%	0.84%
GO:1901617	organic hydroxy compound biosynthetic process	BIOL. PROC.	5.50E-03	5.51E-05	5.00%	2.29%
GO:0010498	proteasomal protein catabolic process	BIOL. PROC.	1.84E-02	2.34E-04	2.97%	1.15%
GO:0009862	systemic acquired resistance, salicylic acid mediated signaling pathway	BIOL. PROC.	2.81E-02	3.83E-04	2.66%	1.01%
GO:0001561	fatty acid alpha-oxidation	BIOL. PROC.	3.83E-02	5.91E-04	0.31%	0.00%
GO:0071705	nitrogen compound transport	BIOL. PROC.	4.23E-02	6.58E-04	11.72%	7.84%
GO:0005788	endoplasmic reticulum lumen	CELL. COMP.	4.92E-10	1.08E-13	2.34%	0.14%
GO:0009505	plant-type cell wall	CELL. COMP.	2.13E-05	5.40E-08	3.91%	1.06%
GO:0005886	plasma membrane	CELL. COMP.	4.49E-05	1.24E-07	20.00%	12.57%
GO:0030126	COPI vesicle coat	CELL. COMP.	6.20E-05	1.80E-07	1.09%	0.06%
GO:0000327	lytic vacuole within protein storage vacuole	CELL. COMP.	8.68E-05	3.25E-07	0.63%	0.01%
GO:0022626	cytosolic ribosome	CELL. COMP.	9.32E-03	9.96E-05	3.13%	1.17%
GO:0005774	vacuolar membrane	CELL. COMP.	2.16E-02	2.79E-04	5.00%	2.47%
GO:0009506	plasmodesma	CELL. COMP.	2.83E-02	3.95E-04	7.03%	4.04%
GO:0003756	protein disulfide isomerase activity	MOL. FUNCT.	1.82E-04	8.43E-07	1.09%	0.07%
GO:0005198	structural molecule activity	MOL. FUNCT.	1.20E-03	9.40E-06	5.00%	2.08%
GO:0005524	ATP binding	MOL. FUNCT.	2.30E-03	2.05E-05	14.53%	9.27%
GO:0004674	protein serine/threonine kinase activity	MOL. FUNCT.	1.28E-02	1.48E-04	6.09%	3.15%
GO:0004842	ubiquitin-protein transferase activity	MOL. FUNCT.	1.58E-02	1.91E-04	3.28%	1.32%
GO:0005509	calcium ion binding	MOL. FUNCT.	1.80E-02	2.26E-04	2.81%	1.05%
GO:0051670	inulinase activity	MOL. FUNCT.	2.83E-02	3.96E-04	0.31%	0.00%
GO:0052861	glucan endo-1,3-beta-glucanase activity, C-3 substituted reducing group	MOL. FUNCT.	4.85E-02	8.24E-04	0.31%	0.00%

GO:0052862	glucan endo-1,4-beta-glucanase activity, C-3 substituted reducing group	MOL. FUNCT.	4.85E-02	8.24E-04	0.31%	0.00%
GO:0051669	fructan beta-fructosidase activity	MOL. FUNCT.	4.85E-02	8.24E-04	0.31%	0.00%

Supplementary Table S9. KEGG annotation of differentially expressed genes (DEGs) in *Brassica napus* that are more highly expressed in *Lm*-inoculated Westar than in inoculated 74-44 BL (scores > 1.0×10^3).

Gene ID	Expression	log2 Fold Change	adjusted *p*-value	Score	KEGG pathway
BnaA06g31440D	866.61	3.55	1.64E-21	6.40E+04	Purine metabolism
BnaC03g07130D	999.98	2.79	1.08E-11	3.06E+04	Amino sugar and nucleotide sugar metabolism
BnaC01g40210D	823.03	2.07	1.70E-10	1.66E+04	Glycolysis / Gluconeogenesis<br>Carbon fixation in photosynthetic organisms
BnaAnng05990D	357.91	2.00	1.07E-15	1.07E+04	Oxidative phosphorylation<br>Photosynthesis
BnaC06g00540D	1114.69	2.18	1.73E-04	9.16E+03	Purine metabolism
BnaC09g40190D	605.15	2.05	1.85E-07	8.36E+03	Purine metabolism
BnaC03g75900D	254.53	2.06	2.54E-13	6.60E+03	Aminoacyl-tRNA biosynthesis
BnaA03g55200D	246.72	2.00	7.34E-13	6.00E+03	Starch and sucrose metabolism

BnaC03g75530D	177.53	2.01	3.18E-15	5.17E+03	Aminoacyl-tRNA biosynthesis
BnaC05g21140D	96.67	3.21	1.27E-15	4.62E+03	Fatty acid biosynthesis<br>Biotin metabolism
BnaA09g04210D	104.44	2.21	1.95E-17	3.86E+03	Purine metabolism<br>Pyrimidine metabolism<br>Thiamine metabolism
BnaA04g04210D	117.64	2.55	1.68E-12	3.53E+03	Terpenoid backbone biosynthesis
BnaA06g25360D	95.16	2.61	4.73E-11	2.56E+03	Purine metabolism
BnaC06g22630D	344.19	2.31	6.91E-04	2.52E+03	Glycerolipid metabolism<br>Glycerophospholipid metabolism
BnaA04g12390D	45.51	2.57	4.50E-15	1.68E+03	Aminoacyl-tRNA biosynthesis
BnaAnng07310D	36.05	3.62	4.70E-12	1.48E+03	Glycolysis / Gluconeogenesis<br>Pentose phosphate pathway<br>Fructose and mannose metabolism<br>Carbon fixation in photosynthetic organisms
BnaC04g49090D	92.73	2.02	2.14E-06	1.06E+03	Amino sugar and nucleotide sugar metabolism

Supplementary Table S10. Genes of *Brassica napus* that are more highly expressed in mock-inoculated cotyledons of 74-44 BL than in those of mock Westar (scores < 2 x 10^4).

Gene name	Expression	log2 Fold Change	Adjusted *p*-value	Score	InterPro ID	Description
BnaC08g39910D	1652.55	-2.16	1.71E-16	-5.62E+04	IPR004938	Xyloglucan fucosyltransferase
BnaA09g48570D	1148.55	-2.16	3.51E-16	-3.83E+04	IPR000008	C2 calcium-dependent membrane targeting
BnaA09g48570D	1148.55	-2.16	3.51E-16	-3.83E+04	IPR008973	C2 calcium/lipid-binding domain, CaLB
BnaA05g22060D	290.14	-2.10	3.25E-42	-2.53E+04	IPR001680	WD40 repeat

BnaA05g22060D	290.14	-2.10	3.25E-42	-2.53E+04	IPR015943	WD40/YVTN repeat-like-containing domain
BnaA05g22060D	290.14	-2.10	3.25E-42	-2.53E+04	IPR017986	WD40-repeat-containing domain
BnaA05g22060D	290.14	-2.10	3.25E-42	-2.53E+04	IPR019775	WD40 repeat, conserved site
BnaA05g22060D	290.14	-2.10	3.25E-42	-2.53E+04	IPR020472	G-protein beta WD-40 repeat
BnaC09g37530D	904.93	-2.40	2.33E-08	-1.65E+04	IPR008811	Raffinose synthase
BnaC09g37530D	904.93	-2.40	2.33E-08	-1.65E+04	IPR013785	Aldolase-type TIM barrel
BnaC09g37530D	904.93	-2.40	2.33E-08	-1.65E+04	IPR017853	Glycoside hydrolase, superfamily
BnaA09g05080D	642.03	-2.34	6.08E-11	-1.54E+04	IPR000008	C2 calcium-dependent membrane targeting
BnaA09g05080D	642.03	-2.34	6.08E-11	-1.54E+04	IPR008973	C2 calcium/lipid-binding domain, CaLB
BnaA09g05080D	642.03	-2.34	6.08E-11	-1.54E+04	IPR018029	C2 membrane targeting protein
BnaC03g03670D	286.50	-2.51	5.41E-19	-1.31E+04	IPR001680	WD40 repeat
BnaC03g03670D	286.50	-2.51	5.41E-19	-1.31E+04	IPR006594	LisH dimerisation motif
BnaC03g03670D	286.50	-2.51	5.41E-19	-1.31E+04	IPR006595	CTLH, C-terminal LisH motif
BnaC03g03670D	286.50	-2.51	5.41E-19	-1.31E+04	IPR015943	WD40/YVTN repeat-like-containing domain
BnaC03g03670D	286.50	-2.51	5.41E-19	-1.31E+04	IPR017986	WD40-repeat-containing domain
BnaC03g03670D	286.50	-2.51	5.41E-19	-1.31E+04	IPR019775	WD40 repeat, conserved site
BnaC03g03670D	286.50	-2.51	5.41E-19	-1.31E+04	IPR020472	G-protein beta WD-40 repeat
BnaA06g01730D	909.50	-2.90	2.36E-05	-1.22E+04	IPR024286	Domain of unknown function DUF3700
BnaA01g28810D	244.27	-3.56	2.28E-12	-1.01E+04	IPR001220	Legume lectin domain
BnaA01g28810D	244.27	-3.56	2.28E-12	-1.01E+04	IPR008985	Concanavalin A-like lectin/glucanases superfamily
BnaA01g28810D	244.27	-3.56	2.28E-12	-1.01E+04	IPR013320	Concanavalin A-like lectin/glucanase, subgroup
BnaA01g28810D	244.27	-3.56	2.28E-12	-1.01E+04	IPR016363	Lectin

Supplementary Table S11. GO term enrichment of DEGs in *Brassica napus* that are more highly expressed in mock-inoculated 74-44 BL than in mock Westar.

GO ID	GO Name	GO Category	FDR	P-Value	% Test	% Reference
GO:0004402	histone acetyltransferase activity	MOL. FUNCT.	1.42E-01	9.69E-04	1.41%	0.09%

Supplementary Table S12. KEGG annotation of differentially expressed genes (DEGs) in *Brassica napus* that are more highly expressed in mock-inoculated 74-44 BL than in mock-inoculated Westar (scores > 1.0×10^3).

Gene ID	Expression	log2 Fold Change	adjusted p-value	Score	KEGG_pathway
BnaAnng15770D	72.68	-4.42	3.71E-25	-7.84E+03	Fructose and mannose metabolism
BnaC03g02050D	204.28	-2.28	2.04E-13	-5.90E+03	Starch and sucrose metabolism
BnaA09g51530D	19.32	-7.62	5.19E-13	-1.81E+03	Fatty acid biosynthesis<br>Fatty acid elongation

Supplementary Table S13. Genes of *Brassica napus* that are more highly expressed in mock-inoculated cotyledons of Westar than in those of 74-44 BL (scores > 8.6×10^3).

Gene name	Expression	log2 Fold Change	adjusted p-value	Score	InterPro ID	Description
BnaA03g55320D	1683.64	3.48	2.20E-20	1.15E+05	IPR004864	Late embryogenesis abundant protein, LEA-14
BnaC05g14950D	13184.04	2.09	1.21E-02	5.28E+04	IPR001938	Thaumatin
BnaA09g14680D	796.92	2.20	3.59E-24	4.11E+04	IPR000767	Disease resistance protein
BnaA09g14680D	796.92	2.20	3.59E-24	4.11E+04	IPR002182	NB-ARC
BnaC08g41540D	623.42	2.37	7.65E-20	2.83E+04	IPR000960	Flavin monooxygenase FMO
BnaC08g41540D	623.42	2.37	7.65E-20	2.83E+04	IPR020946	Flavin monooxygenase-like
BnaC03g59870D	160.11	5.25	2.16E-19	1.57E+04	IPR000719	Protein kinase, catalytic domain
BnaC03g59870D	160.11	5.25	2.16E-19	1.57E+04	IPR001245	Serine-threonine/tyrosine-protein kinase catalytic domain
BnaC03g59870D	160.11	5.25	2.16E-19	1.57E+04	IPR001611	Leucine-rich repeat
BnaC03g59870D	160.11	5.25	2.16E-19	1.57E+04	IPR002290	Serine/threonine- / dual specificity protein kinase, catalytic domain
BnaC03g59870D	160.11	5.25	2.16E-19	1.57E+04	IPR008271	Serine/threonine-protein kinase, active site
BnaC03g59870D	160.11	5.25	2.16E-19	1.57E+04	IPR011009	Protein kinase-like domain
BnaC03g59870D	160.11	5.25	2.16E-19	1.57E+04	IPR013320	Concanavalin A-like lectin/glucanase, subgroup
BnaC03g59870D	160.11	5.25	2.16E-19	1.57E+04	IPR017441	Protein kinase, ATP binding site
BnaC03g59870D	160.11	5.25	2.16E-19	1.57E+04	IPR020635	Tyrosine-protein kinase, catalytic domain
BnaC03g59870D	160.11	5.25	2.16E-19	1.57E+04	IPR024788	Malectin-like carbohydrate-binding domain
BnaA03g32960D	1854.73	2.09	8.96E-05	1.57E+04	IPR003114	Phox-associated domain
BnaA03g32960D	1854.73	2.09	8.96E-05	1.57E+04	IPR013937	Sorting nexin, C-terminal
BnaA03g32960D	1854.73	2.09	8.96E-05	1.57E+04	IPR013996	PX-associated, sorting nexin 13
BnaA05g33670D	404.63	2.59	5.25E-11	1.08E+04	IPR013057	Amino acid transporter, transmembrane

BnaC09g50120D	224.00	2.46	5.03E-17	8.99E+03	IPR004147	UbiB domain
BnaC09g50120D	224.00	2.46	5.03E-17	8.99E+03	IPR011009	Protein kinase-like domain
BnaCnng49630D	2783.81	2.23	3.98E-02	8.68E+03	IPR001128	Cytochrome P450
BnaCnng49630D	2783.81	2.23	3.98E-02	8.68E+03	IPR002401	Cytochrome P450, E-class, group I
BnaCnng49630D	2783.81	2.23	3.98E-02	8.68E+03	IPR017972	Cytochrome P450, conserved site

Supplementary Table S14. GO term enrichment of DEGs in *Brassica napus* that are more highly expressed in mock-inoculated Westar than in mock 74-44 BL.

GO ID	GO Name	GO Category	FDR	*p*-Value	% Test	% Reference
GO:0043531	ADP binding	MOL. FUNCT.	8.84E-03	4.22E-05	3.68%	0.74%
GO:0004497	monooxygenase activity	MOL. FUNCT.	2.22E-02	2.12E-04	3.31%	0.74%

Supplementary Table S15. KEGG annotation of differentially expressed genes (DEGs) in *Brassica napus* that are more highly expressed in mock-inoculated Westar than in mock 74-44 BL (scores >1.0 x 10^3).

Gene ID	Expression	log2 Fold Change	Adjusted *p*-value	Score	KEGG_Pathway
BnaCnng45230D	320.11	3.50	2.14E-04	4.11E+03	Nitrogen metabolism
BnaC09g48850D	353.63	2.53	1.35E-03	2.57E+03	Phenylpropanoid biosynthesis
BnaA03g36540D	238.98	2.24	2.53E-05	2.46E+03	Glycerophospholipid metabolism<br>Ether lipid metabolism
